# Patterns of Evolution and Host Gene Mimicry in Influenza and Other RNA Viruses

**DOI:** 10.1371/journal.ppat.1000079

**Published:** 2008-06-06

**Authors:** Benjamin D. Greenbaum, Arnold J. Levine, Gyan Bhanot, Raul Rabadan

**Affiliations:** 1 BioMaPS Institute, Rutgers University, Piscataway, New Jersey, United States of America; 2 Institute for Advanced Study, Princeton, New Jersey, United States of America; The Pennsylvania State University, United States of America

## Abstract

It is well known that the dinucleotide CpG is under-represented in the genomic DNA of many vertebrates. This is commonly thought to be due to the methylation of cytosine residues in this dinucleotide and the corresponding high rate of deamination of 5-methycytosine, which lowers the frequency of this dinucleotide in DNA. Surprisingly, many single-stranded RNA viruses that replicate in these vertebrate hosts also have a very low presence of CpG dinucleotides in their genomes. Viruses are obligate intracellular parasites and the evolution of a virus is inexorably linked to the nature and fate of its host. One therefore expects that virus and host genomes should have common features. In this work, we compare evolutionary patterns in the genomes of ssRNA viruses and their hosts. In particular, we have analyzed dinucleotide patterns and found that the same patterns are pervasively over- or under-represented in many RNA viruses and their hosts suggesting that many RNA viruses evolve by mimicking some of the features of their host's genes (DNA) and likely also their corresponding mRNAs. When a virus crosses a species barrier into a different host, the pressure to replicate, survive and adapt, leaves a footprint in dinucleotide frequencies. For instance, since human genes seem to be under higher pressure to eliminate CpG dinucleotide motifs than avian genes, this pressure might be reflected in the genomes of human viruses (DNA and RNA viruses) when compared to those of the same viruses replicating in avian hosts. To test this idea we have analyzed the evolution of the influenza virus since 1918. We find that the influenza A virus, which originated from an avian reservoir and has been replicating in humans over many generations, evolves in a direction strongly selected to reduce the frequency of CpG dinucleotides in its genome. Consistent with this observation, we find that the influenza B virus, which has spent much more time in the human population, has adapted to its human host and exhibits an extremely low CpG dinucleotide content. We believe that these observations directly show that the evolution of RNA viral genomes can be shaped by pressures observed in the host genome. As a possible explanation, we suggest that the strong selection pressures acting on these RNA viruses are most likely related to the innate immune response and to nucleotide motifs in the host DNA and RNAs.

## Author Summary

Viruses are obligate intracellular parasites that use different strategies to sequester host cell machinery and avoid the host immune system. In this paper we explore the genomes of viruses that encode their genetic information in single-stranded RNA, a different material than the one used by their hosts (double-stranded DNA). It is interesting to observe that these viruses share some of the host's characteristics. For instance, one of the most underrepresented motifs in the DNA of vertebrates is the dinucleotide CpG. This is commonly thought to be due to methylation and deamination of cytosine residues in this dinucleotide. Surprisingly, the same CpG suppression is observed in vertebrate RNA viruses but not in RNA phages. We show that RNA viruses present similar dinucleotide pressures as their host genes. We find that the influenza A virus, which originated from an avian reservoir and replicated in humans over many generations, evolves to reduce the frequency of CpG dinucleotides mimicking the human genes. Influenza B, which has been in humans longer, exhibits an extremely low CpG dinucleotide content. These observations suggest that the evolution of RNA viruses is shaped by pressures observed in the host genome.

## Introduction

Viruses are infectious agents unable to reproduce in the absence of a host cell. Their survival depends on their ability to enter the host cell, replicate themselves, and, if possible, avoid the host's immune system. In this paper, we study patterns in the genomic sequences of single-stranded RNA viruses and compare them to their host's genome (DNA) and corresponding mRNAs. As shown below, we find that many nucleotide sequence patterns observed in RNA viruses seem to be correlated with and determined by the patterns in the DNA genomes of their host.

Prior to recent advances in sequencing, it was observed that dinucleotide frequencies deviate from what is randomly expected [1],[2]. The most striking example is the suppression of CpG dinucleotides in vertebrates. The most accepted explanation for the suppression of CpG dinucleotides relies on the methylation of CpG nucleotides and subsequent deamination of 5-methylcytosine, making CpG a mutational hotspot [3],[4]. Consistently, in invertebrates such as insects, worms, fungi, and many bacteria, CpG is not under-represented when compared to its expected frequency, in agreement with the fact that these organisms do not methylate the cytosine residue in CpG dinucleotides (ee, among others, [3],[5]). Another explanation suggests that dinucleotide patterns could be related to DNA structural constraints rather than methylation [6]. Since these explanations have been proposed, some observations have eroded this conventional view and suggested that the above mechanisms provide only a partial explanation. In particular, several other facts suggest that the process of methylation and deamination is not sufficient to explain the observed under-representation of CpG dinucleotides in the DNA of vertebrates. Jabbari et al. [7] analyzed the CpG and TpG (CpA) levels in Drosophila, zebra fish and humans, and demonstrated that the amount of CpG deficiency shows no significant correlation with the level of DNA methylation. A CpG under-representation has also been observed in mitochondrial and bacterial DNA [8] even though methylation has not been detected in some of them [9],[10].

Most interestingly, this same phenomenon of CpG dinucleotide under-representation occurs in most vertebrate viruses [11]–[15], independent of the nature of their genome, whether they are RNA or DNA viruses, or whether they replicate in the nucleus or the cytoplasm of a cell. Clearly the methylation of cytosine residues in CpG dinucleotides in DNA and its deamination cannot explain why the RNA viruses of vertebrates have a similar under-representation of these CpG dinucleotides. Moreover, the other DNA oriented explanations do not account for this pressure. This suggests that there is a mechanism for targeting CpG at the RNA level. It has been suggested that this effect is due to a vertebrate evolutionary event, and that vertebrate viruses have been derived from host sources, reflecting this pressure [11]. It has also been suggested that patterns in RNA are recognized by the innate immune system in a similar context to DNA, although this point has often been dismissed [14].

Interestingly, when unmethylated in DNA, CpG is also known to induce an immunostimmulatory response, which could contribute to its suppression (for a review see Ref.[16] and references therein). This is famously triggered by the pattern recogniton receptor Toll-like receptor 9, which recognizes CpGs in a TA context. Similarly, RNase L has a preferences for some particular dinucleotides, such as ApA and UpA in RNA [17],[18]. This probably reflects a way developed by the innate immune system to distinguish self from non-self.

This work analyzes dinucleotide patterns of many RNA viruses and their hosts. We designed several methods to analyze these patterns relating viruses to their hosts, factorizing out different effects related to codon biases or function amino acid constraints. When these viruses crossed to their new host they came under different selective pressures, as reflected in a human specific mutational nucleotide bias previously described [19]. We analyzed the evolution of influenza A as it moved from avian hosts into the human population and replicated for ninety years in human hosts. This work shows a host specific selection pressure against CpG dinucleotides (especially in a location rich in A and T residues) in many ssRNA viruses. We suggest that the most likely explanation in RNA viruses is the existence of an RNA dinucleotide recognition system, probably linked to the innate immune system of the host. This hypothesis is consistent with the observation that influenza viruses from avian sources elicit much greater cytokine responses in cells and animals than do influenza viruses derived from human sources [20]–[22].

## Methods

Our two-tiered strategy for examining dinucleotide occurrence begins with an initial search for whether or not a given dinucleotide is over- or under-represented in a virus due to a selective pressure. There are several methods that have been considered previously in the literature to evaluate dinucleotide patterns (see among others [3],[7],[8],[11],[23],[24]). We consider some of these methods below and, in addition, have designed a method that takes into account possible biases due to the specific amino acid sequence and codon distribution. This is performed using a Monte Carlo method for the coding region of genes. To insure that it is selection that has distorted a dinucleotide frequency in a genome, we identify and eliminate other sources of frequency distortion. Because of the degeneracy of the genetic code, genomes of different organisms and their genes often show a codon bias, preferring to employ one codon over another. Likewise, proteins often employ different levels of amino acids and prefer certain sequences of amino acids for a specific function. This will create dinucleotide patterns correlated with a particular amino acid sequence rather than the pressure for host adaptation. To eliminate or normalize for these constraints, we first explore the sequence space for those possible gene sequences by sampling many different possible sequences using a Monte Carlo type method. This is done by incorporating the constraints for C+G content, codon usage, and amino acid sequence in the coding region of a real gene to generate a number of equivalent sequences in simulation sequence space, as was done analogously in a previous analysis [25]. This method, therefore, explicitly fixes the amino acid distribution and order for the coding region of a given gene, while at the same time preserving the overall frequencies of codons within each amino acid. In this way, the method is essentially equivalent to reshuffling the codons randomly within a given gene's amino acid structure.

According to this method, a set of randomized sequences is generated and compared to the real sequence. A p-value is generated for each possible dinucleotide combination by counting the number of occurrences of that dinucleotide in the third and first positions of sequential codons and comparing this count to the number of dinucleotide occurrences in these positions in the real sequence. The fraction of times the dinucleotide count is less in the real sequences than in the random sequences is the p-value for determining under-representation. The p-values for over-representation were generated using a similar procedure. This allows for a blind search of coding regions for dinucleotide patterns. Once these patterns have been identified, they can then be further examined.

Following the targeting of a dinucleotide as over- or under- represented by assignment of p-values, we further characterize this dinucleotide to search for the causes of this distortion. This is particularly important in the case of influenza, where one wishes to examine a time series. In addition to generating p-values, these randomized sequences were also used to measure the degree to which the total number of dinucleotides are higher or lower than expected. This measure, which is designated by the index *ρ*, is simply the number of times the dinucleotide occurs in the third and first positions of sequential codons in a real sequence divided by the average number of times it occurs in the third and first position of sequential codons for the Monte Carlo sequences generated by that real sequence. Therefore, it is given by,
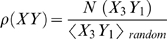
where N(*X*_3_ *Y*_1_) is the number of XY dinucleotides in third and first codon position and <XY> is the mean value from the generated distribution. This quantity was measured over the eight longest open reading frames of the influenza virus, concatenated together to further eliminate pressures due to a particular gene's function. The Monte Carlo procedure requires that the codon frequencies and amino acid sequences be unaltered in computing *ρ* and the p-value. This guarantees that the observed effects are not biased by amino acid structure or codon usage biases of a particular host.

In addition to simple dinucleotide counting, two other indices were used to measure dinucleotide presence. The first is the well-known odds ratio, which we denote by *η*, which is used since the C+G content of the genome can by itself distort the frequencies of dinucleotides. The *η* measure can correct for this explicitly by estimating the expected random frequencies of a dinucleotide for a given C+G value. This is defined as the ratio of the odds of finding a dinucleotide in a given sequence, divided by the product of the odds of finding each nucleotide that forms the pair. Thus,
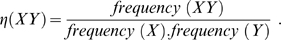
This ratio equals one if the dinucleotide occurs at the amount expected from the nucleotide frequency in the sequence, ie. if X and Y are uncorrelated. Measuring *η* allows us to eliminate the possibility that a pattern is simply due to the frequency of the nucleotides present within a sequence. Therefore, this statistic removes the effect of changes in dinucleotide content due to changes in the underlying nucleotide composition, such as from mutational bias.

However, given that the genetic code is degenerate, we could still have many different nucleotide sequences that code for the same amino acid sequence within a given RNA viral genome. For the special case of CpG, an index that we call the Arginine Index is also used to account for this redundancy. As CpG is present in four of the six codons that represent Arginine, this index, called *r*, is the fraction of times Arginine is comprised of one of the CpG codons. It is defined as,
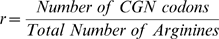
If CpG is actively selected against, one would expect the fraction of times a CpG codon is used to code for Arginine to decline. In the time series of influenza sequences we studied, there is not a discernable reduction in Arginine over time, implying that if a change is observed it is not due to selection against Arginine.

The *ρ* statistic and p-values focus on the coding region, whereas *η* can be used for either coding regions or whole genomes. In the analysis presented below, the *η* statistic was applied to 691 sense ssRNA, 256 antisense ssRNA and 477 dsRNA whole genomes (including independent segments in the case of segmented viruses), to present a broad picture of CpG pressure in these viruses. In this database each viral strain is represented only once. The viruses were obtained from the NCBI genome database (http://www.ncbi.nlm.nih.gov/genomes/VIRUSES/10239.html) and they are listed in Text S2. In Text S3, we selected the viral genomes from purely human hosts. We also applied the *η* statistic to the coding regions of all human and chicken genomes obtained from the University of California Santa Cruz Genome Browser [26]–[29].

The cumulative effect of these observations across many viruses eliminates the possibility that the dinucleotide patterns identified are specific to a particular virus. In our analysis, factors such as codon usage, amino acid structure, and mutational bias were removed by choosing a set of measures, each of which factored out a set of possible causes for the observed pattern. For instance, *η* takes into account possible nucleotide bias, but not amino acid selection or codon usage. The *ρ* index takes into account all of these three factors. The remaining significant dinucleotide patterns that survive these tests should, therefore, have functional or evolutionary causes which require an explanation. To identify a possible explanation, we have applied the measures defined above to study the evolution of influenza A and B in different hosts since 1918. The influenza sequences (674 human H1N1, 24 human H2N2, 1499 human H3N2, 65 human H5N1, 170 influenza B and 969 avian sequences) were obtained from (http://www.ncbi.nlm.nih.gov/genomes/FLU/FLU.html), the Influenza Virus Resource at NCBI. This overlaps with the aforementioned NCBI viral genome database, where each strain is only allotted one representative sequence, for segments from the following five influenza A strains: A/Goose/Guangdong/1/96(H5N1), A/Hong Kong/1073/99(H9N2), A/Puerto Rico/8/34(H1N1), |A/Korea/426/68(H2N2), A/New York/392/2004(H3N2).

## Results

The above methods were initially used to search the coding region of influenza for nucleotide distortion patterns. By comparing the observed nucleotide sequence and dinucleotide frequency in an RNA virus to the generated sample, we obtained the statistical significance of the measured values of the dinucleotide content relative to the null distribution of the same parameters over all possible sequences with the same C+G content, codon frequency and amino acid sequence. We find that the dinucleotides CpG and TpA are highly under-represented and CpA and TpG are over-represented (p-value <10^−3^ for CpA and p-value <10^−4^ for the others). No other dinucleotide patterns were significant at a 95% confidence level. The same result holds in all eight segments of the viral genome and the only dinucleotides that are under-represented consistently within confidence are CpG and TpA. This confirms that influenza viruses generally avoid motifs containing CpGs and TpAs, and prefer those containing to CpAs and TpGs. Importantly, as we have discussed before, this result cannot be explained by the C+G content, codon usage or particular constraints of the amino acid sequence, as these factors are specifically accounted for by the search methods. The suppression of CpG dinucleotides in the DNA of vertebrates is a well-known phenomenon for which the accepted explanation is based on the fact that deamination of 5-methylcytosine in methylated CpG dinucleotides produces a cytosine to thymine mutation at a high frequency that is not efficiently corrected by the DNA editing system. However, this is not the explanation for CpG suppression in the influenza RNA genome.

**Figure 1:**
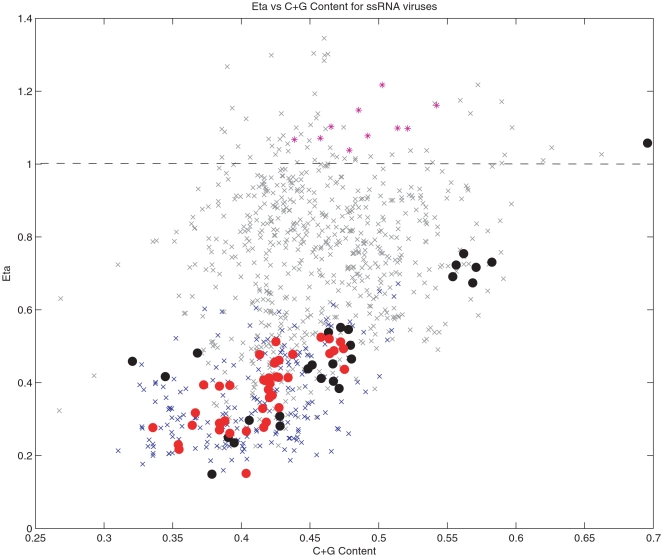
CpG odds ratio (η) versus C+G content for different ssRNA viruses. A value of *η* close to 1 (marked in the figure with a dashed line), means that the measured value is very close to the expected, i.e. no significant pressure to create or eliminate CpGs. Most ssRNA viruses have strong pressure to eliminate CpGs. Human viruses, marked with black (ssRNA+) and red (ssRNA−) filled circles, are at the lower part of the CpG distribution for a given nucleotide content. In particular, the lowest ssRNA viruses in this set are Hepatitis A and Hemagglutinin in influenza C, both human viruses. In contrast, phages do not show any such pressure the remaining viruses, which mostly occur in plants and insects, do not show any significant pressure. All phages listed are ssRNA+.

We find that the same phenomenon of CpG and TpA suppression also occurs in many other vertebrate viruses [11]–[15], independent of the nature of their genome, whether they are RNA or DNA viruses or whether they replicate in the nucleus or cytoplasm. In Figure 1 we summarize the actual and expected CpG frequencies in many different ssRNA viruses as measured by the CpG odds ratio, *η*. A value *η* ∼1 (which is marked by a dashed line), means that the measured value is very close to the expected value and implies the lack of selection pressure (consistent with a null distribution) to create or eliminate CpGs. However, as the figure shows, even after correcting for the expected CpG frequencies, most of the viruses have a strong pressure to eliminate CpGs and this effect is correlated with the host type and the C+G content of the virus. Human viruses in particular, marked with black (ssRNA+) and red (ssRNA−) filled circles, are at the lower part of the CpG frequency distribution for a given C+G content. The lowest ssRNA viruses in this set are the Hepatitis A virus (ssRNA+) and the Hemagglutinin-Esterase encoding gene in the influenza C virus (ssRNA−), both human viruses. The fact that CpG is extremely suppressed in Hepatitis A has been previously observed [11]. Human viruses also show a strong correlation of CpG pressure with C+G content. The black dot on the right of the figure (a very high C+G frequency) is Rubella, which does not show any pressure to eliminate CpGs and has a very high C+G content. In contrast, phages show no CpG pressure, as their value is very close to 1. The majority of the other viruses occur in plants and insects and show lower levels of CpG than in phages, but higher than in humans. Also, most of these many viruses do not show any significant CpG pressure independent of their C+G content. The conclusion from this figure is that ssRNA human viruses, independent of whether they are sense or antisense, have a strong pressure to suppress CpGs that is highly correlated with their C+G content. This effect is completely absent in high C+G viruses like Rubella. Curiously, double stranded RNA viruses (dsRNA) show similar, but not identical, patterns as in the supplemental materials. In particular, cystoviruses (phages) do not suppress CpG dinucleotides and have similar levels as ssRNA+ phages. The human viruses, on the other hand, do present strong pressure to suppress CpGs. The dsRNA virus with the lowest CpG content is a human rotavirus (Rotavirus C, with *η* = 0.4026). However many human dsRNA viruses suppress CpG only slightly, indicating that somewhat different pressures act on these viruses.

**Figure 2:**
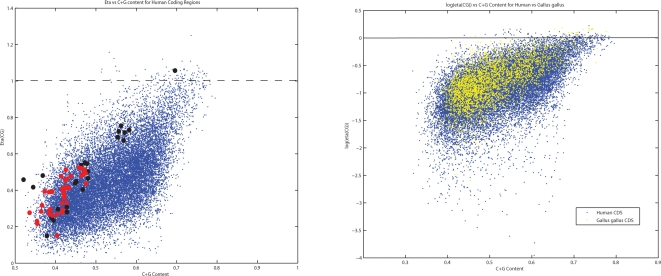
Comparison of CpG suppression in host and viral genomes. Left: CpG odds ratio versus C+G content for human genes in blue. Superimposed on top of these genes are the human RNA viruses from Figure 1, in the same color scheme: Human ssRNA+ (black) and Human ssRNA−(red). Right: Natural logarithm of the CpG odds ratio (log(*η*)) versus C+G content for human (blue) and chicken (yellow) (Gallus gallus) coding genes of at least 500 bases. A value of log(*η*) close to 0 (marked in the figure with a dashed line), indicates that the observed number of CpGs is similar to the expected. CpGs preassures are stronger in human than in birds.

We next examined whether this effect is also present in the host genes. In the left frame of Figure 2, we show the CpG odds ratio *η* versus the C+G content of 22,000 human coding genes (DNA). The values of the odds ratios and their correlations with C+G content show a similar distribution to the one observed in Figure 1 for ssRNA human viruses. If deamination of 5-methylcytosines in DNA is the explanation for CpG suppression in human genes, this must mean that viruses are mimicking the CpG distribution of the host gene's CpG using a different mechanism. If deamination of 5-methylcytosines is not the complete explanation of CpG suppression in human genes, it suggests that there is an additional mechanism for CpG suppression which is common to the host genes and in the RNA of viruses that replicate in these hosts. By exploring the nucleotide sequences that surround these CpG dinucleotides in human genes, it is readily seen that the most over-represented motifs are CpG surrounded by additional cytosine and guanine residues. The most under-represented sequences surrounding these CpG dinucleotides are those rich in adenosine and thymine. For instance, sequences such as ApApCpGpTpT is selected against much more frequently in a DNA genome than is GpGpCpGpCpC [Greenbaum, et al. *in preparation*]. This helps to explain why Rubella, a virus with high C+G content, can survive the pressure to decrease CpGs. By being less likely to possess the above motifs, it therefore would not trigger such an immunostimulatory response. A similar effect, but with opposite correlation, can be observed in relation to the dinucleotide TpA, suggesting that TpA is eliminated in a C+G rich context (summarized in the supplemental materials). It is noteworthy that this seems to contradict the explanation for low TpA in RNA viruses based on particular preferences of RNase L, since, in those cases, RNase L targets UpA in a U+A rich context [30]. Moreover, if this is the cause, one would expect a similar targeting of UpU, as has been observed in enzymatic action on hepatitis C. However, no such suppression was seen in dinucleotide patterns.

**Figure 3:**
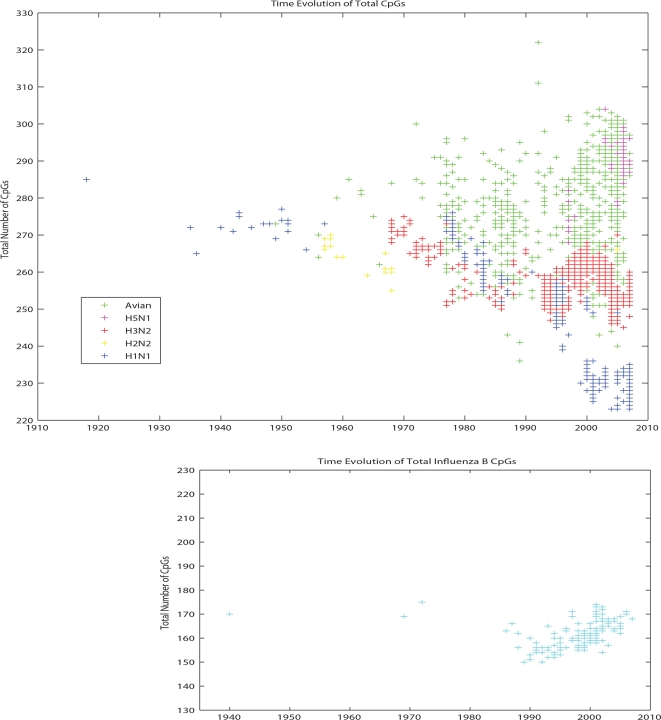
Evolution of the number of CpG motifs in influenza A and B. Top: Evolution of the number of CpGs in influenza A virus in time. When an influenza virus crosses from birds to humans the virus mimics the host genes by reducing the number of CpGs. Bottom: Evolution of the number of CpGs in influenza B virus in time.

Why should these RNA virus genes mimic the DNA of host genes? What happens when an RNA virus from one host with a particular pattern of CpG dinucleotides jumps to another host with a different pattern of dinucleotides? To answer these questions, we explored the movement of influenza viruses from avian hosts to human hosts and followed the evolutionary changes in CpG dinucleotides. In the right hand panel of Figure 2, we plot the natural logarithm of the CpG odds ratio log(*η*) versus C+G content for human (blue) and chicken (red) (Gallus gallus) coding genes (DNA). A value of log(*η*) close to 0 (marked in the figure with a dashed line), indicates that the observed number of CpGs is similar to the expected number based upon a neutral evolution model and corrected for C+G content, codon bias and amino acid content and sequences. Figure 2 demonstrates that selection pressures to eliminate CpGs from the genome are stronger in humans than in birds. For instance, comparing the median value of *η* in human genes versus avian genes, we find that they are significantly different (0.42 vs 0.46) with p-value 4.77×10^−16^ (Mann-Whitney). In Figure 3 we follow the evolutionary changes in CpG content for the influenza A viruses that have jumped from birds to humans three times during the twentieth century. The figure suggests that when an influenza virus crosses from birds to humans, it mimics the DNA host genes by reducing the number of CpGs over time. This effect can be easily appreciated in the evolution of H1N1. H1N1 appeared in humans in 1918, very likely from an avian source [19]. As H1N1 viruses adapted to humans, they reduced the number of CpGs in all eight segments. H2N2 and H3N2 viruses are reassortants with 3 and 2 avian segments, respectably. The more limited loss of CpGs in these two viruses (see Figure 3A) is explained by the blending of human and avian viruses in reassortmant viruses. The extreme case (almost an equilibrium case) of this evolution can be found in influenza B, which most likely entered the human population many centuries ago and is now limited to only a human host. It has adapted to match the human CpG content so completely that it has almost half the number of CpGs as the highest influenza A viruses.

**Figure 4:**
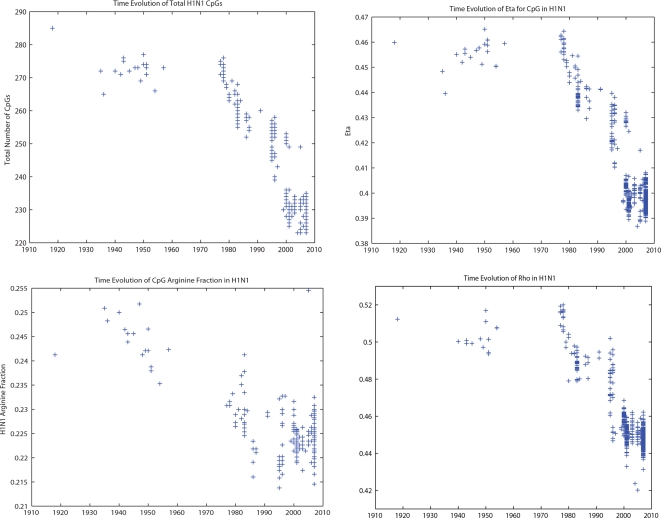
Different measures of dinucleotide suppression applied to the evolution of the human H1N1 virus. Upper left figure: Evolution of the number of CpGs in human H1N1 influenza A virus from 1918–2007. Upper right figure: Evolution of the odds ratio *η* =  CpG/C.G in human H1N1 influenza A virus from 1918–2007. Lower left figure: Evolution of the ratio (r) of CG Arginine (CGN) codons over the total number of arginines (CGN, AGA, AGG) in human H1N1 influenza A virus from 1918–2007. Lower right figure: Evolution of the *ρ* index in human H1N1 influenza A virus from 1918–2007.

Although our Monte Carlo approach controls for codon bias, amino acid frequency and sequence order, all approaches are considered simultaneously when demonstrating whether these variables might cause CpG levels to be under negative selection over evolutionary time scales. To further test this, we have carried out four different kinds of in silico experiments on H1N1, as it presents us with a long and reliable time series. In Figure 4 we show the evolution of four different indexes that quantify these different factors: the total number of CpGs, the odds ratio *η*, the CpG codon usage for arginine and the *ρ* index for CpG. The odds ratio (*η*) factors out the role of possible changes in nucleotide frequences. The CpG codon usage for Arginine measures how often Arginine uses CpG in its codons rather then the other degenerate codons that do not use a CpG but code for Arginine. We can see that this number is very low and gets lower with replication of influenza viruses over evolutionary time scales. The results of these analyses as presented in Figure 4 demonstrate that all these indexes which measure CpG content in influenza viruses decline with time of replication in the human population. This indicates that the CpG suppression is not a result of the amino acid sequence, codon bias or nucleotide mutational biases. Rather, these data suggest that the CpG suppression *determines* the mechanism of mutational biases and codon usage over these evolutionary time scales. Moreover, when these same statistical analyses are applied to TpA (which also starts off at a low value), this analysis shows TpA dinucleotides increasing with replication times in humans. The other statistical measures are weaker for TpA than in CpG dinucleotides, as is shown in Text S1, which indicates that whatever is driving this process is doing so more stringently in regards to CpG than TpA. Thus, though generally under-represented, the presence of TpA is actually *increasing* as it moves from birds to humans and replicates over the time scales shown. Furthermore, when CpA and TpG, the two over-represented dinucleotides initially found by the Monte Carlo, are examined, there is no detectable change in the influenza A genome. In fact, CpG and TpA are the only dinucleotides whose values are detectibly increasing or decreasing by any measure.

## Discussion

We have demonstrated that certain dinucleotides (CpG, TpA, TpG and CpA) are extremely under- or over-represented in influenza and other RNA viruses, independent of factors such as codon frequency and constraints from the amino acid sequence. We have also shown that CpG under-representation is common to many RNA viruses and that it appears correlated with the host. For instance bacteriophages do not show any CpG pressure, while viruses found in humans are consistently the lowest (in ssRNA+, ssRNA− and dsRNA), indicating the greatest selective pressure. This pressure targets ssRNA more strongly and consistently than dsRNA. The suppression is similar to the one observed in the DNA of human genes, indicating that these RNA viruses mimic the host gene's dinucleotide patterns.

We have shown that for the specific case of influenza jumping from avians to humans, it starts decreasing the CpGs content of its RNA genome, mimicking the lower CpG content of human genes compared to avian genes. However a question still remains: why should an RNA virus mimic the host DNA dinucleotide pattern? There are several possible explanations of why these human ssRNA viruses might try to mimic the host pressure to suppress CpGs. One possible explanation is structural [31]–[33]. The most compelling of these explanations is that the difference in body temperature between avain and human hosts alters the dinucleotide content of these RNA viruses in the direction we observe: towards AT and away from GC. However we disfavor this explanation since it does not explain why CpG to TpA is the preferred dinucleotide pattern while all others do not change, and it does not address why CpG is still generally quite low in avian and human viruses. Nor is it clear why the *η* or *ρ* statistics should change under this influence, as they should not be sensitive to mutational bias. The second explanation is that there are deaminating enzymes that act in a preferred dinucleotide context, such as the action of the APOBEC3G enzyme on HIV-1 [34]–[36]. As far as we know, the actions of such enzymes have not been related to ssRNA viruses. Moreover, in this case, the preferred pattern of mutation causes an ApG and ApA enrichment on the positive strand following cytidine deamination on the negative strand [34]. No such pattern was detected for any of the RNA viruses.

After considering the factors listed above, we have come to favor the following hypothesis. Viral RNA genes are selected for or against so as to mimic host mRNA so they can avoid immune detection. This idea has been explored at the protein level in herpesviruses [37] suggesting that these viruses avoid immune detection by mimicry of host epitopes. A reasonable hypothesis that could explain the low content of CpG in RNA viral genomes is that RNA CpG dinucleotides in both the viral genes and the host mRNA are recognized by some components of the innate immune system resulting in the production of cytokines that limit viral replication. In host genes with a high A+T content, CpG dinucleotides are suppressed and this is observed to be the case in many DNA genome viruses that replicate in those hosts [11],[12],[15]. Among the possible explanations for the suppression of CpG dinucleotides in DNA viruses is the presence of methylated cytosine residues in CpG dinucleotides. Not only do these dinucleotides deaminate readily, but their suppression may also be due to the recognition of unmethylated CpG DNA by the Toll-like receptor TLR9, resulting in a higher immune response [38]–[40]. There are two well-known ways by which the innate immune system recognizes foreign CpGs in DNA viruses (see Ref. [40] and references therein for a review of immunostimulatory response to DNA sequences). One is D-type recognition, which releases IFN-gamma. The motifs are stem loops with a CpG in the middle. Most of the motifs have a set of A+T around the CpGs. Another is K-type recognition by TLR9, which stimulates monocytes and activates IL6 and NF-kB. There are several motifs: TCG(A/T)T, i.e. a CpG in the middle and a set of As and Ts. It has been suggested that ssRNA is also recognize by similar CpG motifs [41]. It is known that several proteins (TLR3, TLR7, TLR8, RIG-I, etc) are involved in the recognition of viral RNAs although it is not always clear if there is a specific sequence motif that will engage the receptor and trigger NF-kB transcription of cytokines. In the case of RIG-I, such a molecular signature has been found, but it is unrelated to CpG [42]. The analysis presented here suggests that in an RNA CpG sequence, within a A+T rich context, specific proteins should be looked for that mediate a high cytokine response to that RNA sequence. Interestingly many of the human genes with very low CpG content are precisely the genes involved in this same innate immune response: viz. the IFNAs (2, 5, 6, 14, 16, 17, 21), IL2 and CLECK6A [Greenbaum, et al, *in preparation*]. These innate immune genes have *η* values even lower than any RNA virus. Strikingly, several interferon (IFN2, IFN5, IFN6, IFN14) genes actually have no CpGs in their mRNA whatsoever. Although it is not clear how this phenomenon relates to the selection against CpG motifs in viruses, one could imagine that the production of a large number of mRNAs could trigger a positive feedback loop that destabilizes the innate immune response if the mRNA's CpGs were not highly suppressed.

A final speculation is suggested by an examination of Figure 3, which shows that the original 1918 H1N1 influenza virus and the recent H5N1 avian viruses have a much higher fraction of CpGs in their RNA genome than do those of human adapted influenza viruses. These strains are derived after adaptation to an avian replicative history. If the explanation given here for the selection against motifs containing CpG dinucleotides in ssRNA viruses is correct and the high fraction of these motifs triggers an innate immune response, one would expect a stronger innate immune response in patients infected in the epidemic of 1918 and in present infections of humans with the H5N1 viruses. There are indeed several reports, based both upon the description of the causes of death in the 1918 H1N1 and in the recent H5N1 infections in humans and an experimental analysis of the innate immune response after infections with these viruses to imply that this may indeed be the correct interpretation [20]–[22]. The results presented in this work support this hypothesis and suggest a way to understand why the 1918 influenza virus was so lethal. It also suggests a reason why one might expect a high fatality rate when an avian influenza virus jumps into a human host. Furthermore the selection pressures against the CpG dinucleotides in ssRNA viruses and their variation with the nature of the host, suggest a yet undiscovered mechanism: an ssRNA CpG receptor that triggers lethal levels of cytokines.

Broadly, our results reinforce that understanding the genome patterns of viruses provide a way to interpret and predict viral evolution and give deep insight into the biology of the interaction between a virus and its host.

## Supporting Information

Text S1 Analysis of TpA dinucleotide.
(0.27 MB DOC)

Text S2 List of RNA viral genomes.
(0.14 MB DOC)

Text S3 List of Human RNA viral genomes.
(0.03 MB DOC)
