# Supplementary material for: Patterns of Evolution and Host Gene Mimicry in Influenza and Other RNA Viruses
Source: PLoS Pathog. 2008 Jun 6;4(6):e1000079. doi: 10.1371/journal.ppat.1000079 (PMC2390760; doi:10.1371/journal.ppat.1000079)
Supplement: Text S1 — Analysis of TpA dinucleotide. (0.27 MB DOC) [file ppat.1000079.s001.doc]

**Supplementary Information 1: TA analysis**

In this appendix we summarize the results of the analysis of the TpA dinucleotide pressures.

**Figure 1:** TpA odds ratio (*η*) versus C+G content for different ssRNA viruses. A value of *η* close to 1 (marked in the figure with a dashed line), means that the measured value is very close to the expected, i.e. no significant pressure to create or eliminate TpAs. Human viruses, marked with black (ssRNA+) and red (ssRNA-) filled circles, are at the lower part of the TpA distribution for a given nucleotide content.

**
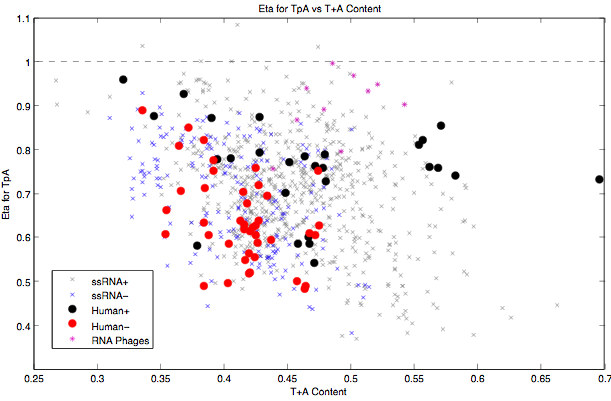
**

**Figure 2:** TpA odds ratio versus C+G content for humans coding regions of at least 500 bases.

**
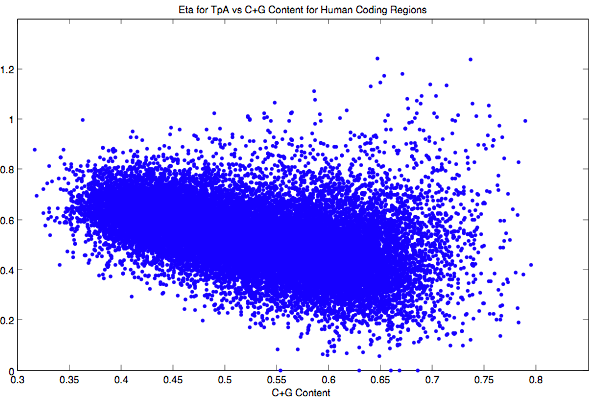
**

**Figure 3: Upper figure:** Evolution of the number of TpAs in influenza B virus in time.

**Lower figure:** Evolution of the number of TpAs in influenza A virus in time.

**Figure 4: Upper left figure:** Evolution of the number of TpAs in human H1N1 influenza A virus from 1918-2007. **Upper right figure:** Evolution of the odds ratio, *η*, for TpA in human H1N1 influenza A virus from 1918-2007. **Lower figure:** Evolution of the *ρ* index in human H1N1 influenza A virus from 1918-2007.
