# Supplementary material for: Patterns of Evolution and Host Gene Mimicry in Influenza and Other RNA Viruses
Source: PLoS Pathog. 2008 Jun 6;4(6):e1000079. doi: 10.1371/journal.ppat.1000079 (PMC2390760; doi:10.1371/journal.ppat.1000079)
Supplement: Text S2 — List of RNA viral genomes. (0.14 MB DOC) [file ppat.1000079.s002.doc]

**Supplementary Information 2: List of RNA viral genomes**

RNA+ viruses:

NC_002640.1| Dengue virus 4, complete genome

NC_001943.1| Human astrovirus, complete genome

NC_001477.1| Dengue virus 1, complete genome

NC_001475.2| Dengue virus 3, complete genome

NC_001474.2| Dengue virus 2, complete genome

NC_005778.1| Chilli veinal mottle virus, complete genome

NC_003602.1| Grapevine virus B, complete genome

NC_002058.3| Poliovirus, complete genome

NC_009825.1| Hepatitis C virus genotype 4, genome

NC_009824.1| Hepatitis C virus genotype 3, genome

NC_008041.1| Redspotted grouper nervous necrosis virus RNA 2, complete sequence

NC_008040.1| Redspotted grouper nervous necrosis virus RNA 1, complete sequence

NC_001812.1| Cymbidium mosaic virus, complete genome

NC_003492.1| Bean yellow mosaic virus, complete genome

NC_001959.2| Norwalk virus, complete genome

NC_010178.1| Pineapple mealybug wilt-associated virus 1, complete genome

NC_009942.1| West Nile virus (lineage I strain NY99), complete genome

NC_007817.1| Enterobacteria phage ID2, complete genome

NC_009992.1| Plum bark necrosis and stem pitting-associated virus, complete genome

NC_009891.1| Seal picornavirus type 1, complete genome

NC_004322.1| Tulip virus X, complete genome

NC_003839.2| Tomato ringspot virus RNA 2, complete sequence

NC_001746.1| Kennedya yellow mosaic virus, complete genome

NC_001671.1| Pea seed-borne mosaic virus, complete genome

NC_001430.1| Human enterovirus D, complete genome

NC_008251.1| Chenopodium mosaic virus X, complete genome

NC_001890.1| Enterobacteria phage Qbeta, complete genome

NC_002700.2| Acinetobacter phage AP205, complete genome

NC_009087.2| Chrysanthemum virus B, complete genome

NC_001563.2| West Nile virus (lineage II strain 956), complete genome

NC_009892.1| Peach chlorotic mottle virus, complete genome

NC_003837.1| Tomato aspermy virus RNA 1, complete sequence

NC_001874.1| Rhopalosiphum padi virus, complete genome

NC_002035.1| Cucumber mosaic virus RNA 2, complete sequence

NC_002034.1| Cucumber mosaic virus RNA 1, complete sequence

NC_001440.1| Cucumber mosaic virus RNA 3, complete sequence

NC_009996.1| Human rhinovirus C, complete genome

NC_009991.1| Phlox Virus B, complete genome

NC_009988.1| Bat coronavirus HKU2, complete genome

NC_009025.1| Israel acute paralysis virus of bees

NC_005854.1| Parietaria mottle virus RNA 3, complete sequence

NC_005028.1| Papaya leaf-distortion mosaic potyvirus, complete genome

NC_004162.2| Chikungunya virus, complete genome

NC_001434.1| Hepatitis E virus, complete genome

NC_009995.1| Moroccan watermelon mosaic virus, complete genome

NC_009994.1| Tobacco vein banding mosaic virus, complete genome

NC_009827.1| Hepatitis C virus genotype 6, complete genome

NC_009826.1| Hepatitis C virus genotype 5, genome

NC_009823.1| Hepatitis C virus genotype 2, complete genome

NC_006551.1| Usutu virus, complete genome

NC_004102.1| Hepatitis C virus, complete genome

NC_003976.2| Ljungan virus, complete genome

NC_001961.1| Porcine reproductive and respiratory syndrome virus, complete genome

NC_001616.1| Potato virus Y, complete genome

NC_001543.1| Rabbit hemorrhagic disease virus-FRG, complete genome

NC_009017.1| Nootka lupine vein-clearing virus, complete genome

NC_006553.1| Duck picornavirus TW90A, complete genome

NC_003628.1| Parsnip yellow fleck virus, complete genome

NC_001441.1| Narcissus mosaic virus, complete genome

NC_003838.1| Tomato aspermy virus RNA 2, complete sequence

NC_003836.1| Tomato aspermy virus RNA 3, complete sequence

NC_009887.1| Human enterovirus 100, complete genome

NC_008311.1| Murine norovirus 1, complete genome

NC_008294.1| Pseudomonas phage PRR1, complete genome

NC_007816.1| Cucumber leaf spot virus, complete genome

NC_005267.1| Raspberry ringspot virus RNA 2, complete sequence

NC_005266.1| Raspberry ringspot virus RNA 1, complete sequence

NC_004718.3| SARS coronavirus, complete genome

NC_003742.1| Cocksfoot streak virus, complete genome

NC_003462.1| Apple stem pitting virus, complete genome

NC_002306.2| Transmissible gastroenteritis virus, complete genome

NC_001785.1| Papaya ringspot virus, complete genome

NC_001472.1| Human enterovirus B, complete genome

NC_001445.1| Plum pox virus, complete genome

NC_001489.1| Hepatitis A virus, complete genome

NC_003693.1| Beet ringspot virus RNA 1, complete sequence

NC_001361.2| Potato virus M, complete genome

NC_001801.1| Cucumber green mottle mosaic virus, complete genome

NC_008250.1| Duck hepatitis virus 1, complete genome

NC_007919.2| Nora virus, complete genome

NC_004124.1| Sweet potato chlorotic stunt virus RNA 2, complete sequence

NC_004123.1| Sweet potato chlorotic stunt virus RNA 1, complete sequence

NC_001639.1| Lactate dehydrogenase-elevating virus, complete genome

NC_009805.1| Wheat eqlid mosaic virus, complete genome

NC_002040.1| Peanut stunt virus RNA 3, complete sequence

NC_002039.1| Peanut stunt virus RNA 2, complete sequence

NC_002038.1| Peanut stunt virus RNA 1, complete sequence

NC_008558.1| Blackberry virus Y, complete genome

NC_009764.1| Coleus vein necrosis virus, complete genome

NC_009759.1| Potato rough dwarf virus, complete genome

NC_009758.1| Marine RNA virus JP-B, complete genome

NC_009757.1| Marine RNA virus JP-A, complete genome

NC_009756.1| Marine RNA virus SOG, complete genome

NC_009750.1| Duck hepatitis virus AP, complete genome

NC_009741.1| Basella rugose mosaic virus, complete genome

NC_003397.1| Bean common mosaic virus, complete genome

NC_001455.2| Potato virus X, complete genome

NC_009745.1| Banana bract mosaic virus, complete genome

NC_009744.1| Wild tomato mosaic virus, complete genome

NC_009743.1| Peace lily mosaic virus, complete genome

NC_009742.1| Telosma mosaic virus, complete genome

NC_009544.1| Solenopsis invicta virus 2, complete genome

NC_007518.1| Heterocapsa circularisquama RNA virus, complete genome

NC_011452.1| Foot-and-mouth disease virus SAT 3, complete genome

NC_011451.1| Foot-and-mouth disease virus SAT 1, complete genome

NC_011450.1| Foot-and-mouth disease virus A, complete genome

NC_004915.1| Foot-and-mouth disease virus Asia 1, complete genome

NC_004004.1| Foot-and-mouth disease virus O, complete genome

NC_003992.2| Foot-and-mouth disease virus SAT 2, complete genome

NC_003045.1| Bovine coronavirus, complete genome

NC_002554.1| Foot-and-mouth disease virus C, complete genome

NC_001617.1| Human rhinovirus 89, complete genome

NC_001490.1| Human rhinovirus B, complete genome

NC_009530.1| Brevicoryne brassicae picorna-like virus, complete genome

NC_008708.2| Strawberry necrotic shock virus RNA 1, complete sequence

NC_008183.1| Black raspberry necrosis virus RNA2, complete sequence

NC_008182.1| Black raspberry necrosis virus RNA1, complete sequence

NC_009693.1| Bat SARS CoV Rp3/2004, complete genome

NC_005876.1| Kakugo virus, complete genome

NC_009696.1| Bat SARS CoV Rm1/2004, complete genome

NC_009695.1| Bat SARS CoV Rf1/2004, complete genome

NC_009694.1| Bat coronavirus HKU3, complete genome

NC_001846.1| Murine hepatitis virus strain A59, complete genome

NC_009657.1| Bat coronavirus 512/2005, complete genome

NC_009642.1| Bell pepper mottle tobamovirus, complete genome

NC_009448.1| Saffold virus, complete genome

NC_006852.1| Murine hepatitis virus strain JHM, complete genome

NC_005831.2| Human coronavirus NL63, complete genome

NC_001786.1| Barmah Forest virus, complete genome

NC_001451.1| Avian infectious bronchitis virus, complete genome

NC_006558.1| Getah virus, complete genome

NC_003480.1| Apple mosaic virus RNA 3, complete sequence

NC_003465.1| Apple mosaic virus RNA 2, complete sequence

NC_003464.1| Apple mosaic virus RNA 1, complete sequence

NC_007289.1| Potato virus S, complete genome

NC_001897.1| Human parechovirus, genome

NC_001409.1| Apple chlorotic leaf spot virus, complete genome

NC_009538.1| Citrus variegation virus RNA2, complete sequence

NC_009537.1| Citrus variegation virus RNA1, complete sequence

NC_009536.1| Citrus variegation virus RNA3, complete sequence

NC_009533.1| Maize white line mosaic virus, complete genome

NC_009532.1| Okra mosaic virus, complete genome

NC_008585.1| Raspberry mottle virus, complete genome

NC_003899.1| Eastern equine encephalitis virus, complete genome

NC_002692.1| Tomato mosaic virus, complete genome

NC_001556.1| Tobacco mild green mosaic virus, complete genome

NC_001437.1| Japanese encephalitis virus, genome

NC_004067.1| Pepino mosaic virus, complete genome

NC_008552.1| Narcissus symptomless virus, complete genome

NC_008366.1| Strawberry chlorotic fleck associated virus, complete genome

NC_008315.1| Bat coronavirus (BtCoV/133/2005), complete genome

NC_008029.1| Homalodisca coagulata virus-1, complete genome

NC_008020.1| Daphne virus S, complete genome

NC_007983.1| Lisianthus necrosis virus, complete genome

NC_007679.1| Nerine virus X, complete genome

NC_007408.1| Alstroemeria virus x, complete genome

NC_007192.1| Lily virus X, complete genome

NC_006962.1| Blackberry yellow vein-associated virus RNA1, complete sequence

NC_006943.1| Hydrangea ringspot virus, complete genome

NC_006568.1| Fragaria chiloensis latent virus RNA 3, complete sequence

NC_006554.1| Sapovirus C12 strain C12

NC_006269.1| Sapovirus Hu/Dresden/pJG-Sap01/DE, complete genome

NC_005138.1| Lily symptomless virus, complete genome

NC_009032.1| Tomato torrado virus RNA2, complete sequence

NC_009013.1| Tomato torrado virus RNA1, complete sequence

NC_008516.1| White bream virus, complete genome

NC_007916.1| Newbury agent 1 virus, complete genome

NC_006875.1| Calicivirus isolate TCG, complete genome

NC_006559.1| Solenopsis invicta virus 1, complete genome

NC_005029.1| Onion yellow dwarf virus, complete genome

NC_004751.1| Cereal yellow dwarf virus-RPV, complete genome

NC_004750.1| Barley yellow dwarf virus - PAV, complete genome

NC_004573.1| Peru tomato mosaic virus, complete genome

NC_004426.1| Wild potato mosaic virus, complete genome

NC_004301.1| Enterobacteria phage FI, complete genome

NC_004146.1| Flock house virus RNA 1, complete sequence

NC_003508.1| Beet soil-borne mosaic virus RNA4, complete sequence

NC_003507.1| Beet soil-borne mosaic virus RNA3, complete sequence

NC_003506.1| Beet soil-borne mosaic virus RNA1, complete sequence

NC_003503.1| Beet soil-borne mosaic virus RNA 2, complete sequence

NC_003499.1| Blueberry scorch virus, complete genome

NC_003377.1| Maize dwarf mosaic virus, complete genome

NC_002795.1| Aconitum latent virus, complete genome

NC_002552.1| Hop latent virus, complete genome

NC_009383.1| Phlox virus S, complete genome

NC_009041.1| Rehmannia mosaic virus, complete genome

NC_009029.1| Kokobera virus, complete genome

NC_009028.1| Ilheus virus, complete genome

NC_009027.1| Iguape virus, complete genome

NC_009026.1| Bussuquara virus, complete genome

NC_008719.1| Sepik virus, complete genome

NC_008718.1| Entebbe bat virus, complete genome

NC_008707.1| Strawberry necrotic shock virus RNA 2, complete sequence

NC_008706.1| Strawberry necrotic shock virus RNA 3, complete sequence

NC_008604.1| Culex flavivirus, complete genome

NC_008393.1| Pepper severe mosaic virus, complete genome

NC_008310.1| Hibiscus latent Singapore virus, complete genome

NC_008292.1| Passiflora latent carlavirus, complete genome

NC_008039.1| Prune dwarf virus RNA 1, complete sequence

NC_008038.1| Prune dwarf virus, complete genome

NC_008037.1| Prune dwarf virus RNA 2, complete sequence

NC_008028.1| Daphne virus Y, complete genome

NC_007913.1| Konjak mosaic virus, complete genome

NC_007729.1| Maize necrotic streak virus, complete genome

NC_007728.1| East Asian Passiflora virus, complete genome

NC_007619.1| Kelp fly virus, complete genome

NC_007609.1| Dulcamara mottle virus, complete genome

NC_007522.1| Schizochytrium single-stranded RNA virus, complete genome

NC_007433.1| Shallot yellow stripe virus, complete genome

NC_007415.1| Sclerotinia sclerotiorum debilitation-associated RNA virus, complete genome

NC_007216.1| Wisteria vein mosaic virus, complete genome

NC_007180.1| Thunberg fritillary virus, complete genome

NC_007147.1| Pennisetum mosaic virus, complete genome

NC_007025.1| Feline coronavirus, complete genome

NC_006965.1| Strawberry latent ringspot virus RNA2, complete sequence

NC_006964.1| Strawberry latent ringspot virus RNA1, complete sequence

NC_006963.1| Blackberry yellow vein virus RNA2, complete sequence

NC_006948.1| Mint virus X, complete genome

NC_006947.1| Karshi virus, complete genome

NC_006944.1| Mint virus 1, complete genome

NC_006941.1| Cucumber vein yellowing virus, complete genome

NC_006939.1| Olive mild mosaic virus, complete genome

NC_006567.1| Fragaria chiloensis latent virus RNA 2, complete sequence

NC_006566.1| Fragaria chiloensis latent virus RNA 1, complete sequence

NC_006550.1| Sweet potato chlorotic fleck virus, complete genome

NC_006494.1| Varroa destructor virus 1, complete genome

NC_006264.1| Gremmeniella abietina mitochondrial RNA virus S2, complete genome

NC_006262.1| Watermelon mosaic virus, complete genome

NC_003975.2| Patchouli mild mosaic virus RNA 1, complete sequence

NC_003792.2| Cycas necrotic stunt virus RNA 2, complete sequence

NC_003790.1| Chicken astrovirus, complete genome

NC_003786.2| Satsuma dwarf virus RNA 2, complete sequence

NC_003785.2| Satsuma dwarf virus RNA 1, complete sequence

NC_003687.1| Powassan virus, complete genome

NC_003627.1| Maize chlorotic mottle virus, complete genome

NC_003510.1| Beet virus Q RNA 1, complete sequence

NC_003215.1| Semliki forest virus, complete genome

NC_002568.2| Sesbania mosaic virus, complete genome

NC_002036.1| Pea early browning virus RNA 1, complete sequence

NC_001918.1| Aichi virus, complete genome

NC_001841.1| Sweet potato feathery mottle virus, complete genome

NC_001749.2| Apple stem grooving virus, complete genome

NC_002031.1| Yellow fever virus, complete genome

NC_002027.1| Brome mosaic virus RNA 2, complete sequence

NC_002026.1| Brome mosaic virus RNA 1, complete sequence

NC_002025.1| Alfalfa mosaic virus RNA 3, complete sequence

NC_002024.2| Alfalfa mosaic virus RNA 2, complete sequence

NC_001495.1| Alfalfa mosaic virus RNA 1, complete sequence

NC_001461.1| Bovine viral diarrhea virus 1, complete genome

NC_001428.1| Human enterovirus C, complete genome

NC_001368.1| Pea early browning virus RNA 2, complete sequence

NC_001366.1| Theilovirus, complete genome

NC_003630.1| Pepper mild mottle virus, complete genome

NC_001333.1| Enterobacteria phage fr, complete genome

NC_003514.1| Beet necrotic yellow vein virus RNA 1, complete sequence

NC_007731.1| Alternanthera mosaic virus, complete genome

NC_002028.1| Brome mosaic virus RNA 3, complete sequence

NC_004452.3| Beet black scorch virus, complete genome

NC_002532.2| Equine arteritis virus, complete genome

NC_001545.1| Rubella virus, complete genome

NC_005210.1| Beet pseudo-yellows virus RNA 2, complete sequence

NC_005209.1| Beet pseudo-yellows virus RNA 1, complete sequence

NC_009021.1| Bat coronavirus HKU9-1, complete genome

NC_006271.1| Cherry rasp leaf virus, complete genome

NC_001504.1| Melon necrotic spot virus, genome

NC_009020.1| Bat coronavirus HKU5-1, complete genome

NC_009019.1| Bat coronavirus HKU4-1, complete genome

NC_005304.1| Beet mosaic virus, complete genome

NC_003747.1| Ryegrass mottle virus, complete genome

NC_003491.1| Beet mild yellowing virus, complete genome

NC_003436.1| Porcine epidemic diarrhea virus, complete genome

NC_002509.2| Turnip mosaic virus, complete genome

NC_007733.1| Angelonia flower break virus, complete genome

NC_008824.1| Narcissus degeneration virus, complete genome

NC_007580.2| St. Louis encephalitis virus, complete genome

NC_008716.1| Maracuja mosaic virus, complete genome

NC_008715.1| Possum enterovirus W6, complete genome

NC_008714.1| Possum enterovirus W1, complete genome

NC_003375.1| Garlic virus A, complete genome

NC_003224.1| Zucchini yellow mosaic virus, complete genome

NC_001612.1| Human enterovirus A, complete genome

NC_003604.2| Grapevine virus A, complete genome

NC_008614.1| Cucumber mottle virus, complete genome

NC_004422.1| Youcai mosaic virus, complete genome

NC_008580.1| Rabbit vesivirus, complete genome

NC_010624.1| Sapovirus Mc10, complete genome

NC_003982.1| Equine rhinitis A virus, complete genome

NC_000940.1| Porcine enteric calicivirus, complete genome

NC_001544.1| Ross River virus, complete genome

NC_001481.2| Feline calicivirus, complete genome

NC_005092.1| Ectropis obliqua picorna-like virus, complete genome

NC_008169.1| Cytoplasmic citrus leprosis virus RNA-1, complete sequence

NC_006272.1| Cherry rasp leaf virus RNA2, complete sequence

NC_006057.1| Arabis mosaic virus RNA 1, complete sequence

NC_006056.1| Arabis mosaic virus RNA 2, complete sequence

NC_006063.1| Potato yellow vein virus, complete genome

NC_006061.1| Potato yellow vein virus segment 3, complete sequence

NC_005287.1| Johnsongrass chlorotic stripe mosaic virus, complete genome

NC_006062.1| Potato yellow vein virus, complete genome

NC_004144.1| Flock house virus, complete genome

NC_003608.1| Hibiscus chlorotic ringspot virus, complete genome

NC_001822.1| Leek white stripe virus, complete genome

NC_001721.1| Olive latent virus 1, complete genome

NC_001339.1| Artichoke mottled crinkle virus, complete genome

NC_008266.1| Narcissus common latent virus, complete genome

NC_007341.1| Tomato chlorosis virus RNA 2, complete sequence

NC_007340.1| Tomato chlorosis virus RNA 1, complete sequence

NC_005285.1| Pelargonium necrotic spot virus, complete genome

NC_004830.2| Deformed wing virus, complete genome

NC_004012.1| Garlic virus E, complete genome

NC_004011.1| Leek yellow stripe virus, complete genome

NC_003983.1| Equine rhinitis B virus 1, complete genome

NC_003930.1| Salmon pancreas disease virus, complete genome

NC_003878.1| Zucchini green mottle mosaic virus, complete genome

NC_003610.1| Kyuri green mottle mosaic virus, complete genome

NC_003557.1| Garlic latent virus, complete genome

NC_003400.1| Scallion virus X, complete genome

NC_003399.1| Scallion mosaic virus, complete genome

NC_003347.1| Grapevine fleck virus, complete genome

NC_000939.1| Pothos latent virus, complete genome

NC_001818.1| Galinsoga mosaic virus, complete genome

NC_001728.1| Odontoglossum ringspot virus, complete genome

NC_001411.2| Black beetle virus, complete genome

NC_001555.1| Tobacco etch virus, complete genome

NC_004421.1| Bovine kobuvirus, complete genome

NC_008365.1| Streptocarpus flower break virus, complete genome

NC_003005.1| Taura syndrome virus, complete genome

NC_003800.1| Squash mosaic virus RNA 2, complete sequence

NC_002657.1| Classical swine fever virus, complete genome

NC_008295.1| Penstemon ringspot virus, complete genome

NC_005849.1| Parietaria mottle virus RNA 2, complete sequence

NC_005848.1| Parietaria mottle virus RNA 1, complete sequence

NC_008170.1| Cytoplasmic citrus leprosis virus RNA-2, complete sequence

NC_007001.1| Cassia yellow blotch virus RNA3, complete sequence

NC_007000.1| Cassia yellow blotch virus RNA2, complete sequence

NC_006999.1| Cassia yellow blotch virus RNA1, complete sequence

NC_005039.1| Yokose virus, complete genome

NC_004995.1| Pea stem necrosis virus, complete genome

NC_004106.1| Paprika mild mottle virus, complete genome

NC_003852.1| Obuda pepper virus, complete genome

NC_003782.1| Himetobi P virus, complete genome

NC_003781.1| Infectious flacherie virus, complete genome

NC_003779.1| Plautia stali intestine virus, complete genome

NC_003449.1| Striped Jack nervous necrosis virus, complete genome

NC_003376.1| Garlic virus C, complete genome

NC_003355.1| Crucifer tobamovirus, complete genome

NC_002187.1| Japanese iris necrotic ring virus, complete genome

NC_001859.1| Bovine enterovirus, complete genome

NC_003606.1| Johnsongrass mosaic virus, complete genome

NC_003845.1| Tobacco streak virus RNA 3, complete sequence

NC_003807.1| Sweet clover necrotic mosaic virus RNA 2, complete sequence

NC_003806.1| Sweet clover necrotic mosaic virus RNA 1, complete sequence

NC_003741.1| Red clover mottle virus RNA 1, complete sequence

NC_003740.1| Raspberry bushy dwarf virus RNA 2, complete sequence

NC_003739.1| Raspberry bushy dwarf virus RNA 1, complete sequence

NC_003738.1| Red clover mottle virus RNA 2, complete sequence

NC_003632.1| Potato aucuba mosaic virus, complete genome

NC_008249.1| Chickpea chlorotic stunt virus, complete genome

NC_007732.1| Porcine hemagglutinating encephalomyelitis virus, complete genome

NC_006577.2| Human coronavirus HKU1, complete genome

NC_006066.1| Humulus japonicus latent virus, complete genome

NC_006065.1| Humulus japonicus latent virus, complete genome

NC_006064.1| Humulus japonicus latent virus, complete genome

NC_003517.1| Beet necrotic yellow vein virus RNA 4, complete sequence

NC_003516.1| Beet necrotic yellow vein virus RNA 3, complete sequence

NC_003515.1| Beet necrotic yellow vein virus RNA 2, complete sequence

NC_003513.1| Beet necrotic yellow vein virus RNA 5, complete sequence

NC_005286.1| Pelargonium flower break virus, complete genome

NC_001632.1| Rice tungro spherical virus, complete genome

NC_004346.1| Subterranean clover mottle virus, complete genome

NC_003481.1| Barley stripe mosaic virus RNA 2, complete sequence

NC_003478.1| Barley stripe mosaic virus RNA 3, complete sequence

NC_003469.1| Barley stripe mosaic virus RNA 1, complete sequence

NC_001517.1| Pepper mottle virus, complete genome

NC_003417.1| Mayaro virus, complete genome

NC_002792.1| Ribgrass mosaic virus, complete genome

NC_001710.1| GB virus C/Hepatitis G virus, complete genome

NC_001479.1| Encephalomyocarditis virus, complete genome

NC_001948.1| Rupestris stem pitting associated virus-1, complete genome

NC_004053.1| Ophiostoma mitovirus 5, complete genome

NC_004052.1| Ophiostoma mitovirus 4, complete genome

NC_004049.1| Ophiostoma mitovirus 3a, complete genome

NC_003603.1| Groundnut rosette virus, complete genome

NC_001748.1| Papaya mosaic virus, complete genome

NC_003853.1| Pea enation mosaic virus-2, complete genome

NC_004145.1| Boolarra virus RNA 2, complete sequence

NC_001726.1| Carrot mottle mimic virus, complete genome

NC_004054.1| Ophiostoma novo-ulmi mitovirus 6-Ld, complete genome

NC_007448.1| Grapevine leafroll-associated virus 2, complete genome

NC_007447.1| Breda virus, complete genome

NC_007017.1| Pelargonium line pattern virus, complete genome

NC_006950.1| Citrus sudden death-associated virus, complete genome

NC_006946.1| Apricot pseudo-chlorotic leaf spot virus, complete genome

NC_006265.1| Carrot red leaf virus, complete genome

NC_006060.1| Opuntia virus X, complete genome

NC_006059.1| Zygocactus virus X, complete genome

NC_005985.1| Pelargonium chlorotic ring pattern virus, complete genome

NC_005904.1| Hordeum mosaic virus, complete genome

NC_005903.1| Agropyron mosaic virus, complete genome

NC_005899.1| Dendrolimus punctatus tetravirus RNA2, complete sequence

NC_005898.1| Dendrolimus punctatus tetravirus RNA1, complete sequence

NC_005896.1| Strawberry pallidosis associated virus RNA 2, complete sequence

NC_005895.1| Strawberry pallidosis associated virus RNA 1, complete sequence

NC_005819.1| Sclerophthora macrospora virus A RNA 3, complete sequence

NC_005818.1| Sclerophthora macrospora virus A RNA 2, complete sequence

NC_005817.1| Sclerophthora macrospora virus A RNA 1, complete sequence

NC_005790.1| Turkey astrovirus 2, complete genome

NC_005343.1| Poplar mosaic virus, complete genome

NC_005290.1| Broad bean wilt virus 1 RNA 2, complete sequence

NC_005289.1| Broad bean wilt virus 1 RNA 1, complete sequence

NC_005288.1| Lily mottle virus, complete genome

NC_005281.1| Heterosigma akashiwo RNA virus SOG263, complete genome

NC_005147.1| Human coronavirus OC43, complete genome

NC_005136.1| Oat necrotic mottle virus, complete genome

NC_005132.1| Botrytis virus X, complete genome

NC_005097.1| Tobacco ringspot virus RNA 1, complete sequence

NC_005096.1| Tobacco ringspot virus RNA 2, complete sequence

NC_005095.1| Macrobrachium rosenbergii nodavirus RNA-2, complete sequence

NC_005094.1| Macrobrachium rosenbergii nodavirus RNA-1, complete sequence

NC_005065.1| Little cherry virus 2, complete genome

NC_005064.1| Kamiti River virus, complete genome

NC_005062.1| Omsk hemorrhagic fever virus, complete genome

NC_004810.1| Cucurbit yellow stunting disorder virus RNA2, complete sequence

NC_004809.1| Cucurbit yellow stunting disorder virus RNA1, complete sequence

NC_004807.1| Kashmir bee virus, complete genome

NC_004756.1| Beet western yellows virus, complete genome

NC_004752.1| Yam mosaic virus, complete genome

NC_004730.1| Indian peanut clump virus RNA 2, complete sequence

NC_004729.1| Indian peanut clump virus RNA 1, complete sequence

NC_004725.1| Cucumber Bulgarian virus, complete genome

NC_004724.1| Grapevine rootstock stem lesion associated virus, complete genome

NC_004723.1| Pear latent virus, complete genome

NC_004667.1| Grapevine leafroll-associated virus 3, complete genome

NC_004666.1| Barley yellow dwarf virus - GAV, complete genome

NC_004579.1| Mink astrovirus, complete genome

NC_004560.1| Oyster mushroom spherical virus, complete genome

NC_004553.1| Turnip rosette virus, complete genome

NC_004542.1| Canine calicivirus, complete genome

NC_004541.1| Walrus calicivirus, complete genome

NC_004451.1| Simian picornavirus 1, complete genome

NC_004441.1| Porcine enterovirus B, complete genome

NC_004440.1| Tomato black ring virus RNA 2, complete sequence

NC_004439.1| Tomato black ring virus RNA 1, complete sequence

NC_004425.1| Broad bean necrosis virus RNA 3, complete sequence

NC_004424.1| Broad bean necrosis virus RNA 2, complete sequence

NC_004423.1| Broad bean necrosis virus RNA 1, complete sequence

NC_004366.1| Tobacco bushy top virus, complete genome

NC_004365.1| Aphid lethal paralysis virus, complete genome

NC_004364.1| Prunus necrotic ringspot virus RNA 3, complete sequence

NC_004363.1| Prunus necrotic ringspot virus RNA2, complete sequence

NC_004362.1| Prunus necrotic ringspot virus RNA1, complete sequence

NC_004355.1| Alkhurma virus, complete genome

NC_004142.1| Boolarra virus RNA1, complete sequence

NC_004137.1| Epinephelus tauvina nervous necrosis virus RNA 1, complete sequence

NC_004136.1| Epinephelus tauvina nervous necrosis virus RNA 2, complete sequence

NC_004122.1| Spring beauty latent virus RNA 3, complete sequence

NC_004121.1| Spring beauty latent virus RNA 2, complete sequence

NC_004120.1| Spring beauty latent virus RNA 1, complete sequence

NC_004119.1| Montana myotis leukoencephalitis virus, complete genome

NC_004064.1| Calicivirus strain NB, complete genome

NC_004063.1| Turnip yellow mosaic virus, complete genome

NC_004060.1| Southern bean mosaic virus, complete genome

NC_004051.1| Saccharomyces cerevisiae narnavirus 20S RNA <W>, complete genome

NC_004050.1| Saccharomyces cerevisiae narnavirus 23S RNA <T>, complete genome

NC_004047.1| Bean common mosaic necrosis virus, complete genome

NC_004046.1| Cryphonectria parasitica mitovirus 1-NB631, complete genome

NC_004045.1| Beet western yellows ST9 associated virus, complete genome

NC_004039.1| Potato virus A, complete genome

NC_004035.1| Sorghum mosaic virus, complete genome

NC_004017.1| Oat mosaic virus RNA 2, complete sequence

NC_004016.1| Oat mosaic virus RNA 1, complete sequence

NC_004015.1| Sorghum chlorotic spot virus RNA 2, complete sequence

NC_004014.1| Sorghum chlorotic spot virus RNA 1, complete sequence

NC_004013.1| Cowpea aphid-borne mosaic virus, complete genome

NC_004010.1| Potato virus V, complete genome

NC_004008.1| Broad bean mottle virus RNA 1, complete sequence

NC_004007.1| Broad bean mottle virus RNA 2, complete sequence

NC_004006.1| Broad bean mottle virus RNA 3, complete sequence

NC_003990.1| Avian encephalomyelitis virus, complete genome

NC_003988.1| Simian enterovirus A, complete genome

NC_003987.1| Porcine enterovirus A, complete genome

NC_003985.1| Porcine teschovirus 1, complete genome

NC_003974.1| Patchouli mild mosaic virus RNA 2, complete sequence

NC_003924.1| Cricket paralysis virus, complete genome

NC_003908.1| Western equine encephalomyelitis virus, complete genome

NC_003900.1| Aura virus, complete genome

NC_003877.1| Citrus leaf blotch virus, complete genome

NC_003870.1| Sugarcane striate mosaic associated virus, complete genome

NC_003849.1| Plantago asiatica mosaic virus, complete genome

NC_003844.1| Tobacco streak virus RNA 1, complete sequence

NC_003842.1| Tobacco streak virus RNA 2, complete sequence

NC_003840.1| Tomato ringspot virus RNA 1, complete sequence

NC_003835.1| Tulare apple mosaic virus RNA3, complete sequence

NC_003834.1| Tulare apple mosaic virus RNA2, complete sequence

NC_003833.1| Tulare apple mosaic virus RNA1, complete sequence

NC_003821.2| Turnip crinkle virus, complete genome

NC_003820.1| White clover mosaic virus, complete genome

NC_003811.1| Tobacco rattle virus RNA 2, complete sequence

NC_003810.1| Spinach latent virus RNA 3, complete sequence

NC_003809.1| Spinach latent virus RNA 2, complete sequence

NC_003808.1| Spinach latent virus RNA 1, complete sequence

NC_003805.1| Tobacco rattle virus RNA 1, complete sequence

NC_003799.1| Squash mosaic virus RNA 1, complete sequence

NC_003797.1| Sweet potato mild mottle virus, complete genome

NC_003795.1| Shallot virus X, complete genome

NC_003794.1| Strawberry mild yellow edge virus, complete genome

NC_003791.1| Cycas necrotic stunt virus RNA 1, complete sequence

NC_003788.1| Apple latent spherical virus segment 2, complete sequence

NC_003787.1| Apple latent spherical virus segment 1, complete sequence

NC_003784.1| Black queen cell virus, complete genome

NC_003783.1| Triatoma virus, complete genome

NC_003780.1| Acyrthosiphon pisum virus, complete genome

NC_003775.1| Red clover necrotic mosaic virus RNA 2, complete sequence

NC_003756.1| Red clover necrotic mosaic virus RNA 1, complete sequence

NC_003743.1| Turnip yellows virus, complete genome

NC_003725.1| Potato mop-top virus RNA 2, complete sequence

NC_003724.1| Potato mop-top virus RNA 3, complete sequence

NC_003723.1| Potato mop-top virus RNA 1, complete sequence

NC_003694.1| Beet ringspot virus RNA 2, complete sequence

NC_003692.1| Pariacoto virus RNA2, complete sequence

NC_003691.1| Pariacoto virus RNA1, complete sequence

NC_003690.1| Langat virus, complete genome

NC_003689.1| Cherry virus A, complete genome

NC_003688.1| Cucurbit aphid-borne yellows virus, complete genome

NC_003680.1| Barley yellow dwarf virus - MAV, complete genome

NC_003679.1| Border disease virus 1, complete genome

NC_003678.1| Pestivirus Giraffe-1, complete genome

NC_003677.1| Pestivirus Reindeer-1, complete genome

NC_003674.1| Olive latent virus 2 RNA 2, complete sequence

NC_003673.1| Olive latent virus 2 RNA 1, complete sequence

NC_003672.1| Peanut clump virus RNA 1, complete sequence

NC_003671.1| Olive latent virus 2 RNA 3, complete sequence

NC_003670.1| Pepper ringspot virus RNA 2, complete sequence

NC_003669.1| Pepper ringspot virus RNA 1, complete sequence

NC_003668.1| Peanut clump virus RNA 2, complete sequence

NC_003651.1| Pelargonium zonate spot virus RNA 3, complete sequence

NC_003650.1| Pelargonium zonate spot virus RNA 2, complete sequence

NC_003649.1| Pelargonium zonate spot virus RNA 1, complete sequence

NC_003634.1| Physalis mottle virus, complete genome

NC_003633.1| Oat chlorotic stunt virus, complete genome

NC_003629.1| Pea enation mosaic virus-1, complete genome

NC_003626.1| Maize chlorotic dwarf virus, complete genome

NC_003623.1| Grapevine fanleaf virus RNA 2, complete sequence

NC_003622.1| Grapevine chrome mosaic virus RNA 1, complete sequence

NC_003621.1| Grapevine chrome mosaic virus RNA 2, complete sequence

NC_003618.1| Lettuce infectious yellows virus RNA 2, complete sequence

NC_003617.1| Lettuce infectious yellows virus RNA 1, complete sequence

NC_003615.1| Grapevine fanleaf virus RNA 1, complete sequence

NC_003605.1| Lettuce mosaic virus, complete genome

NC_003570.1| Elm mottle virus RNA 3, complete sequence

NC_003569.1| Elm mottle virus RNA 1, complete sequence

NC_003568.1| Elm mottle virus RNA 2, complete sequence

NC_003550.1| Cowpea mosaic virus RNA 2, complete sequence

NC_003549.1| Cowpea mosaic virus RNA 1, complete sequence

NC_003548.1| Citrus leaf rugose virus RNA 1, complete sequence

NC_003547.1| Citrus leaf rugose virus RNA 2, complete sequence

NC_003546.1| Citrus leaf rugose virus RNA 3, complete sequence

NC_003545.1| Cowpea severe mosaic virus RNA 1, complete sequence

NC_003544.1| Cowpea severe mosaic virus RNA 2, complete sequence

NC_003543.1| Cowpea chlorotic mottle virus RNA 1, complete sequence

NC_003542.1| Cowpea chlorotic mottle virus RNA 3, complete sequence

NC_003541.1| Cowpea chlorotic mottle virus RNA 2, complete sequence

NC_003537.1| Dasheen mosaic virus, complete genome

NC_003536.1| Clover yellow vein virus, complete genome

NC_003535.1| Cowpea mottle virus, complete genome

NC_003532.1| Cymbidium ringspot virus, complete genome

NC_003531.1| Carnation ringspot virus RNA 2, complete sequence

NC_003530.1| Carnation ringspot virus RNA 1, complete sequence

NC_003520.1| Beet soil-borne virus RNA 1, complete sequence

NC_003519.1| Beet soil-borne virus RNA 3, complete sequence

NC_003518.1| Beet soil-borne virus RNA 2, complete sequence

NC_003512.1| Beet virus Q RNA 3, complete sequence

NC_003511.1| Beet virus Q RNA 2, complete sequence

NC_003509.1| Blackcurrant reversion virus RNA1, complete sequence

NC_003502.1| Blackcurrant reversion virus RNA 2, complete sequence

NC_003501.1| Brome streak mosaic virus, complete genome

NC_003500.1| Carnation Italian ringspot virus, complete genome

NC_003496.1| Bean pod mottle virus RNA 1, complete sequence

NC_003495.1| Bean pod mottle virus RNA 2, complete sequence

NC_003487.1| Tobacco necrosis virus D, complete genome

NC_003483.1| Barley mild mosaic virus RNA 1, complete sequence

NC_003482.1| Barley mild mosaic virus RNA2, complete sequence

NC_003453.1| American plum line pattern virus RNA3, complete sequence

NC_003452.1| American plum line pattern virus RNA2, complete sequence

NC_003451.1| American plum line pattern virus RNA1, complete sequence

NC_003448.1| Striped Jack nervous necrosis virus RNA1, complete sequence

NC_003446.1| Strawberry mottle virus RNA2, complete sequence

NC_003445.1| Strawberry mottle virus RNA 1, complete sequence

NC_003433.1| Sleeping disease virus, complete genome

NC_003412.1| Euprosterna elaeasa virus, complete genome

NC_003398.1| Sugarcane mosaic virus, complete genome

NC_003369.1| Bean leafroll virus, complete genome

NC_003113.1| Perina nuda picorna-like virus, complete genome

NC_003093.1| Indian citrus ringspot virus, complete genome

NC_003092.1| Simian hemorrhagic fever virus, complete genome

NC_003077.1| Equine rhinovirus 3, complete genome

NC_003056.1| Soybean dwarf virus, complete genome

NC_003004.1| Broad bean wilt virus 2 RNA2, complete sequence

NC_003003.1| Broad bean wilt virus 2 RNA1, complete sequence

NC_002991.1| Barley yellow mosaic virus RNA 2, complete sequence

NC_002990.1| Barley yellow mosaic virus RNA 1, complete sequence

NC_002815.2| Cactus virus X, complete genome

NC_002786.1| Maize rayado fino virus, complete genome

NC_002766.1| Beet chlorosis virus, complete genome

NC_002729.1| Banana mild mosaic virus, complete genome

NC_002691.1| Nodamura virus RNA2, complete sequence

NC_002690.1| Nodamura virus RNA1, complete sequence

NC_002645.1| Human coronavirus 229E, complete genome

NC_002634.1| Soybean mosaic virus, complete genome

NC_002633.1| Cucumber fruit mottle mosaic virus, complete genome

NC_002618.2| Cocksfoot mottle virus, complete genome

NC_002615.1| European brown hare syndrome virus, complete genome

NC_002604.1| Botrytis virus F, complete genome

NC_002600.1| Peanut mottle virus, complete genome

NC_002598.1| Panicum mosaic virus, complete genome

NC_002588.1| Chayote mosaic virus, complete genome

NC_002551.1| Vesicular exanthema of swine virus, complete genome

NC_002548.1| Acute bee paralysis virus, complete genome

NC_002500.1| Cherry mottle leaf virus, complete genome

NC_002470.1| Turkey astrovirus, complete genome

NC_002469.1| Ovine astrovirus, complete genome

NC_002468.1| Cherry necrotic rusty mottle virus, complete genome

NC_002359.1| Chinese wheat mosaic virus RNA1, complete sequence

NC_002358.1| Oat golden stripe virus RNA 1, complete sequence

NC_002357.1| Oat golden stripe virus RNA 2, complete sequence

NC_002356.1| Chinese wheat mosaic virus RNA2, complete sequence

NC_002351.1| Soil-borne cereal mosaic virus RNA1, complete sequence

NC_002350.1| Wheat yellow mosaic virus RNA 1, complete sequence

NC_002349.1| Wheat yellow mosaic virus RNA 2, complete sequence

NC_002330.1| Soil-borne cereal mosaic virus RNA2, complete sequence

NC_002250.1| Enterobacteria phage KU1, complete genome

NC_002198.2| Cereal yellow dwarf virus-RPS, complete genome

NC_002164.1| Poinsettia mosaic virus, complete genome

NC_002160.2| Barley yellow dwarf virus-PAS, complete genome

NC_001278.1| Diaporthe ambigua RNA virus 1, complete genome

NC_001265.1| Carnation mottle virus, complete genome

NC_000947.1| Japanese yam mosaic virus, complete genome

NC_000943.1| Murray Valley encephalitis virus, complete genome

NC_000874.1| Sugarcane yellow leaf virus, complete genome

NC_002066.1| Sacbrood virus, complete genome

NC_002042.1| Soil-borne wheat mosaic virus RNA2, complete sequence

NC_002041.1| Soil-borne wheat mosaic virus RNA1, complete sequence

NC_002037.1| Black beetle virus RNA 2, complete sequence

NC_001982.1| Helicoverpa armigera stunt virus RNA 2, complete sequence

NC_001981.1| Helicoverpa armigera stunt virus RNA 1, complete sequence

NC_001990.1| Nudaurelia capensis beta virus, complete genome

NC_001977.1| Erysimum latent virus, complete genome

NC_001946.1| Cherry green ring mottle virus, complete genome

NC_001886.1| Wheat streak mosaic virus, complete genome

NC_001873.1| Turnip vein-clearing virus, complete genome

NC_001837.1| Hepatitis GB virus A, complete genome

NC_001836.1| Little cherry virus 1, complete genome

NC_001834.1| Drosophila C virus, complete genome

NC_002032.1| Bovine viral diarrhea virus genotype 2, complete genome

NC_001814.1| Ryegrass mosaic virus, complete genome

NC_001809.1| Louping ill virus, complete genome

NC_001800.1| Garlic virus X, complete genome

NC_001793.1| Oat blue dwarf virus, complete genome

NC_001780.1| Saguaro cactus virus, complete genome

NC_001777.1| Tobacco necrosis virus A, complete genome

NC_001768.1| Tobacco vein mottling virus, complete genome

NC_001753.1| Clover yellow mosaic virus, complete genome

NC_001747.1| Potato leafroll virus, complete genome

NC_001696.1| Lucerne transient streak virus, complete genome

NC_001672.1| Tick-borne encephalitis virus, complete genome

NC_001661.1| Citrus tristeza virus, complete genome

NC_001658.1| Cassava common mosaic virus, complete genome

NC_001655.1| Hepatitis GB virus B, complete genome

NC_001642.1| Bamboo mosaic virus, complete genome

NC_001633.1| Mushroom bacilliform virus, complete genome

NC_001628.1| Pseudomonas phage PP7, complete genome

NC_001625.1| Southern cowpea mosaic virus, complete genome

NC_001600.1| Cardamine chlorotic fleck virus, complete genome

NC_001598.1| Beet yellows virus, complete genome

NC_001575.1| Rice yellow mottle virus, complete genome

NC_001564.1| Cell fusing agent virus, complete genome

NC_001554.1| Tomato bushy stunt virus, complete genome

NC_001547.1| Sindbis virus, complete genome

NC_001513.1| Ononis yellow mosaic virus, complete genome

NC_001512.1| O'nyong-nyong virus, complete genome

NC_001483.1| Foxtail mosaic virus, complete genome

NC_001480.1| Eggplant mosaic virus, complete genome

NC_001469.1| Cucumber necrosis virus, complete genome

NC_001449.1| Venezuelan equine encephalitis virus, complete genome

NC_001426.1| Enterobacteria phage GA, complete genome

NC_001417.1| Enterobacterio phage MS2, complete genome

NC_001367.1| Tobacco mosaic virus, complete genome

NC_003996.1| Tamana bat virus, genome

NC_003676.1| Apoi virus, genome

NC_003675.1| Rio Bravo virus, genome

NC_003635.1| Modoc virus, genome

ssRNA- viruses:

NC_010253.1| Allpahuayo virus segment S, complete sequence

NC_010247.1| Amapari virus segment S, complete sequence

NC_010256.1| Bear Canyon virus segment S, complete sequence

NC_010255.1| Bear Canyon virus segment L, complete sequence

NC_010254.1| Cupixi virus segment S, complete sequence

NC_010252.1| Cupixi virus segment L, complete sequence

NC_010251.1| Amapari virus segment L, complete sequence

NC_010250.1| Oliveros virus segment L, complete sequence

NC_010249.1| Allpahuayo virus segment L, complete sequence

NC_010248.1| Oliveros virus segment S, complete sequence

NC_005894.1| Pirital virus segment S, complete sequence

NC_006317.1| Sabia virus segment S, complete sequence

NC_006313.1| Sabia virus segment L, complete sequence

NC_005897.1| Pirital virus segment L, complete sequence

NC_005082.1| Guanarito virus segment L, complete sequence

NC_005081.1| Junin virus segment S, complete sequence

NC_005080.1| Junin virus segment L, complete sequence

NC_005079.1| Machupo virus segment L, complete sequence

NC_005078.1| Machupo virus segment S, complete sequence

NC_005077.1| Guanarito virus segment S, complete sequence

NC_002617.1| Newcastle disease virus, complete genome

NC_001796.2| Human parainfluenza virus 3, complete genome

NC_006318.1| Toscana virus segment S, complete sequence

NC_005220.1| Uukuniemi virus segment M, complete sequence

NC_003619.1| Groundnut bud necrosis virus segment S, complete sequence

NC_005214.1| Uukuniemi virus, complete genome

NC_002051.1| Tomato spotted wilt virus genomic RNA, segment S, complete sequence

NC_002052.1| Tomato spotted wilt virus RNA L, complete sequence

NC_004161.1| Reston Ebola virus, complete genome

NC_002803.1| Spring viremia of carp virus, complete genome

NC_002549.1| Zaire ebolavirus, complete genome

NC_001608.3| Lake Victoria marburgvirus - Musoke, complete genome

NC_001542.1| Rabies virus, complete genome

NC_003620.1| Groundnut bud necrosis virus segment M, complete sequence

NC_009896.1| Akabane virus segment S, complete sequence

NC_009895.1| Akabane virus segment M, complete sequence

NC_009894.1| Akabane virus segment L, complete sequence

NC_001552.1| Sendai virus, complete genome

NC_006320.1| Toscana virus segment M, complete sequence

NC_006319.1| Toscana virus segment L, complete sequence

NC_006505.1| Infectious salmon anemia virus segment 1, complete sequence

NC_006503.1| Infectious salmon anemia virus

NC_006502.1| Infectious salmon anemia virus

NC_006501.1| Infectious salmon anemia virus

NC_006500.1| Infectious salmon anemia virus

NC_006499.1| Infectious salmon anemia virus segment 6, complete sequence

NC_006498.1| Infectious salmon anemia virus segment 7, complete sequence

NC_006497.1| Infectious salmon anemia virus segment 8, complete sequence

NC_007364.1| Influenza A virus (A/Goose/Guangdong/1/96(H5N1)) segment 8, complete sequence

NC_007363.1| Influenza A virus (A/Goose/Guangdong/1/96(H5N1)) strain A/Goose/Guangdong/1/96(H5N1)

NC_001560.1| Vesicular stomatitis Indiana virus, complete genome

NC_009640.1| Porcine rubulavirus, complete genome

NC_007362.1| Influenza A virus (A/Goose/Guangdong/1/96(H5N1)) segment 4, complete sequence

NC_007361.1| Influenza A virus (A/Goose/Guangdong/1/96(H5N1)) strain A/Goose/Guangdong/1/96(H5N1)

NC_009609.1| Orchid fleck virus RNA 2, complete sequence

NC_009608.1| Orchid fleck virus RNA 1, complete sequence

NC_009489.1| Mapuera virus, complete genome

NC_006508.1| Thogoto virus segment 1, complete sequence

NC_006507.1| Thogoto virus segment 5, complete sequence

NC_006506.1| Thogoto virus segment 4, complete sequence

NC_006504.1| Thogoto virus segment 6, complete sequence

NC_006496.1| Thogoto virus segment 3, complete sequence

NC_006495.1| Thogoto virus segment 2, complete sequence

NC_006316.1| Citrus psorosis virus RNA3, complete sequence

NC_006315.1| Citrus psorosis virus RNA2, complete sequence

NC_006314.1| Citrus psorosis virus RNA1, complete sequence

NC_005238.1| Seoul virus strain Seoul 80-39 clone 1

NC_004294.1| Lymphocytic choriomeningitis virus segment S, complete sequence

NC_004291.1| Lymphocytic choriomeningitis virus segment L, complete sequence

NC_006306.1| Influenza C virus, complete genome

NC_004905.2| Influenza A virus (A/Hong Kong/1073/99(H9N2)), complete genome

NC_009528.1| European bat lyssavirus 2, complete genome

NC_009527.1| European bat lyssavirus 1, complete genome

NC_004110.1| La Crosse virus segment S, complete sequence

NC_004109.1| La Crosse virus segment M, complete sequence

NC_004108.1| La Crosse virus segment L, complete sequence

NC_003776.1| Rice stripe virus RNA 3, complete sequence

NC_003755.1| Rice stripe virus RNA 1, complete sequence

NC_003754.1| Rice stripe virus RNA 2, complete sequence

NC_003753.1| Rice stripe virus RNA 4, complete sequence

NC_003468.2| Andes virus segment L, complete sequence

NC_003467.2| Andes virus segment M, complete sequence

NC_003466.1| Andes virus segment S, complete sequence

NC_003043.1| Avian paramyxovirus 6, complete genome

NC_001927.1| Bunyamwera virus, complete genome

NC_008514.1| Siniperca chuatsi rhabdovirus, complete genome

NC_006437.1| Hantavirus Z10 segment M, complete sequence

NC_006435.1| Hantavirus Z10 segment L, complete sequence

NC_006433.1| Hantavirus Z10 segment S, complete sequence

NC_006428.1| Simian virus 41, complete genome

NC_005235.1| Dobrava-Belgrade virus strain DOBV/Ano-Poroia/Afl9/1999

NC_005234.1| Dobrava virus segment M, complete sequence

NC_005233.1| Dobrava virus segment S, complete sequence

NC_005228.1| Tula virus segment M, complete sequence

NC_005227.1| Tula virus segment S, complete sequence

NC_004907.1| Influenza A virus (A/Hong Kong/1073/99(H9N2)) segment 7, complete sequence

NC_004906.1| Influenza A virus (A/Hong Kong/1073/99(H9N2)) segment 8, complete sequence

NC_002020.1| Influenza A virus (A/Puerto Rico/8/34(H1N1)) segment 8, complete sequence

NC_002016.1| Influenza A virus (A/Puerto Rico/8/34(H1N1)) segment 7, complete sequence

NC_007903.1| Mobala virus segment S, complete sequence

NC_007652.1| Avian metapneumovirus

NC_006942.1| Taro vein chlorosis virus, complete genome

NC_006572.1| Mopeia Lassa reassortant 29 segment L, complete sequence

NC_006447.1| Pichinde virus, complete genome

NC_005300.2| Crimean-Congo hemorrhagic fever virus segment M, complete sequence

NC_004297.1| Lassa virus segment L, complete sequence

NC_002251.1| Northern cereal mosaic virus, complete genome

NC_002043.1| Rift Valley fever virus L segment, complete sequence

NC_001926.1| Bunyamwera virus M segment, complete sequence

NC_005302.1| Crimean-Congo hemorrhagic fever virus segment S, complete sequence

NC_006573.1| Mopeia Lassa reassortant 29 segment S, complete sequence

NC_004296.1| Lassa virus segment S, complete sequence

NC_005224.1| Puumala virus segment S, complete sequence

NC_005219.1| Hantaan virus segment M, complete sequence

NC_005218.1| Hantaan virus segment S, complete sequence

NC_001615.2| Sonchus yellow net virus

NC_005237.1| Seoul virus segment M, complete sequence

NC_005236.1| Seoul virus strain 80-39 segment S, complete sequence

NC_005225.1| Puumala virus segment L, complete sequence

NC_005223.1| Puumala virus segment M, complete sequence

NC_005222.1| Hantaan virus segment L, complete sequence

NC_002045.1| Rift Valley fever virus segment S, complete sequence

NC_002044.2| Rift Valley fever virus M segment, complete sequence

NC_001607.1| Borna disease virus, complete genome

NC_005301.3| Crimean-Congo hemorrhagic fever virus segment L, complete sequence

NC_001498.1| Measles virus, complete genome

NC_007642.1| Lettuce necrotic yellows virus, complete genome

NC_006383.2| Peste-des-petits-ruminants virus, complete genome

NC_006296.2| Rinderpest virus (strain Kabete O), complete genome

NC_005283.1| Dolphin morbillivirus, complete genome

NC_000855.1| Viral hemorrhagic septicemia virus, complete genome

NC_001925.1| Bunyamwera virus L segment, complete sequence

NC_004148.2| Human metapneumovirus, complete genome

NC_008307.1| Melon yellow spot virus segment M, complete sequence

NC_008306.1| Melon yellow spot virus segment L, complete sequence

NC_008303.1| Capsicum chlorosis virus segment M, complete sequence

NC_008302.1| Capsicum chlorosis virus segment L, complete sequence

NC_008301.1| Capsicum chlorosis virus segment S, complete sequence

NC_008300.1| Melon yellow spot virus segment S, complete sequence

NC_003843.1| Watermelon spotted wilt virus segment S, complete sequence

NC_003841.1| Watermelon spotted wilt virus segment M, complete sequence

NC_003832.1| Watermelon spotted wilt virus segment L, complete sequence

NC_003625.1| Impatiens necrotic spot virus segment L, complete sequence

NC_003624.1| Impatiens necrotic spot virus segment S, complete sequence

NC_003616.1| Impatiens necrotic spot virus segment M, complete sequence

NC_003614.1| Groundnut bud necrosis virus segment L, complete sequence

NC_002050.1| Tomato spotted wilt virus RNA M, complete sequence

NC_006312.1| Influenza C virus (C/Ann Arbor/1/50) segment 6, complete sequence

NC_006311.1| Influenza C virus (C/Ann Arbor/1/50) segment 5, complete sequence

NC_006310.1| Influenza C virus (C/Ann Arbor/1/50) segment 4, complete sequence

NC_006309.1| Influenza C virus (C/Ann Arbor/1/50) segment 3, complete sequence

NC_006308.1| Influenza C virus (C/Ann Arbor/1/50) segment 2, complete sequence

NC_005221.1| Uukuniemi virus segment S, complete sequence

NC_003746.1| Rice yellow stunt virus, complete genome

NC_002200.1| Mumps virus, complete genome

NC_007906.1| Ippy virus segment L, complete sequence

NC_007905.1| Ippy virus segment S, complete sequence

NC_007904.1| Mobala virus segment L, complete sequence

NC_005975.1| Maize mosaic virus, complete genome

NC_006575.1| Mopeia virus AN20410 segment S, complete sequence

NC_006574.1| Mopeia virus AN20410 segment L, complete sequence

NC_007803.1| Beilong virus, complete genome

NC_007620.1| Menangle virus, complete genome

NC_007454.1| J-virus, complete genome

NC_007382.1| Influenza A virus (A/Korea/426/68(H2N2)) segment 6, complete sequence

NC_007381.1| Influenza A virus (A/Korea/426/68(H2N2)) segment 5, complete sequence

NC_007380.1| Influenza A virus (A/Korea/426/68(H2N2)) segment 8, complete sequence

NC_007378.1| Influenza A virus (A/Korea/426/68(H2N2)) segment 1, complete sequence

NC_007377.1| Influenza A virus (A/Korea/426/68(H2N2)) segment 7, complete sequence

NC_007376.1| Influenza A virus (A/Korea/426/68(H2N2)) segment 3, complete sequence

NC_007375.1| Influenza A virus (A/Korea/426/68(H2N2)) segment 2, complete sequence

NC_007374.1| Influenza A virus (A/Korea/426/68(H2N2)) segment 4, complete sequence

NC_007373.1| Influenza A virus (A/New York/392/2004(H3N2)) segment 1, complete sequence

NC_007372.1| Influenza A virus (A/New York/392/2004(H3N2)) segment 2, complete sequence

NC_007371.1| Influenza A virus (A/New York/392/2004(H3N2)) segment 3, complete sequence

NC_007370.1| Influenza A virus (A/New York/392/2004(H3N2)) segment 8, complete sequence

NC_007369.1| Influenza A virus (A/New York/392/2004(H3N2)) segment 5, complete sequence

NC_007368.1| Influenza A virus (A/New York/392/2004(H3N2)) segment 6, complete sequence

NC_007367.1| Influenza A virus (A/New York/392/2004(H3N2)) segment 7, complete sequence

NC_007366.1| Influenza A virus (A/New York/392/2004(H3N2)) segment 4, complete sequence

NC_007360.1| Influenza A virus (A/Goose/Guangdong/1/96(H5N1)) segment 5, complete sequence

NC_007359.1| Influenza A virus (A/Goose/Guangdong/1/96(H5N1)) segment 3, complete sequence

NC_007358.1| Influenza A virus (A/Goose/Guangdong/1/96(H5N1)) segment 2, complete sequence

NC_007357.1| Influenza A virus (A/Goose/Guangdong/1/96(H5N1)) segment 1, complete sequence

NC_007020.1| Tupaia rhabdovirus, complete genome

NC_006579.1| Pneumonia virus of mice J3666, complete genome

NC_006439.1| Pichinde virus segment L, complete sequence

NC_006432.1| Sudan ebolavirus, complete genome

NC_006430.1| Simian parainfluenza virus 5, complete genome

NC_006429.1| Mokola virus, complete genome

NC_006054.1| Lettuce ring necrosis virus RNA 4, complete sequence

NC_006053.1| Lettuce ring necrosis virus RNA 3, complete sequence

NC_006052.1| Lettuce ring necrosis virus RNA 2, complete sequence

NC_006051.1| Lettuce ring necrosis virus RNA 1, complete sequence

NC_005974.1| Maize fine streak virus, complete genome

NC_005777.1| Oropouche virus segment S, complete sequence

NC_005776.1| Oropouche virus segment L, complete sequence

NC_005775.1| Oropouche virus segment M, complete sequence

NC_005339.1| Mossman virus, complete genome

NC_005226.1| Tula virus segment L, complete sequence

NC_005217.1| Sin Nombre virus segment L, complete sequence

NC_005216.1| Sin Nombre virus segment S, complete sequence

NC_005215.1| Sin Nombre virus segment M, complete sequence

NC_005093.1| Hirame rhabdovirus, complete genome

NC_005084.2| Fer-de-lance virus, complete genome

NC_005036.1| Goose paramyxovirus SF02, complete genome

NC_004912.1| Influenza A virus (A/Hong Kong/1073/99(H9N2)) segment 3, complete sequence

NC_004911.1| Influenza A virus (A/Hong Kong/1073/99(H9N2)) segment 2, complete sequence

NC_004910.1| Influenza A virus (A/Hong Kong/1073/99(H9N2)) segment 1, complete sequence

NC_004909.1| Influenza A virus (A/Hong Kong/1073/99(H9N2)) segment 6, complete sequence

NC_004908.1| Influenza A virus (A/Hong Kong/1073/99(H9N2)) segment 4, complete sequence

NC_004782.1| Mirafiori lettuce virus RNA 3, complete sequence

NC_004781.1| Mirafiori lettuce virus RNA 2, complete sequence

NC_004780.1| Mirafiori lettuce virus RNA 4, complete sequence

NC_004779.1| Mirafiori lettuce virus RNA 1, complete sequence

NC_004293.1| Tacaribe virus segment S, complete sequence

NC_004292.1| Tacaribe virus segment L, complete sequence

NC_004159.1| Dugbe virus segment L, complete sequence

NC_004158.1| Dugbe virus segment M, complete sequence

NC_004157.1| Dugbe virus segment S, complete sequence

NC_004074.1| Tioman virus, complete genome

NC_003461.1| Human parainfluenza virus 1 strain Washington/1964, complete genome

NC_003443.1| Human parainfluenza virus 2, complete genome

NC_003243.1| Australian bat lyssavirus, complete genome

NC_002728.1| Nipah virus, complete genome

NC_002526.1| Bovine ephemeral fever virus, complete genome

NC_002328.1| Rice grassy stunt virus RNA 6, complete sequence

NC_002327.1| Rice grassy stunt virus RNA 5, complete sequence

NC_002326.1| Rice grassy stunt virus RNA 4, complete sequence

NC_002325.1| Rice grassy stunt virus RNA 3, complete sequence

NC_002324.1| Rice grassy stunt virus RNA 2, complete sequence

NC_002323.1| Rice grassy stunt virus RNA 1, complete sequence

NC_002211.1| Influenza B virus RNA 8, complete sequence

NC_002210.1| Influenza B virus RNA 7, complete sequence

NC_002209.1| Influenza B virus RNA 6, complete sequence

NC_002208.1| Influenza B virus RNA 5, complete sequence

NC_002207.1| Influenza B virus RNA 4, complete sequence

NC_002206.1| Influenza B virus RNA-3, complete sequence

NC_002205.1| Influenza B virus RNA-2, complete sequence

NC_002204.1| Influenza B virus RNA 1, complete sequence

NC_002199.1| Tupaia paramyxovirus, complete genome

NC_002161.1| Bovine parainfluenza virus 3, complete genome

NC_000903.1| Snakehead rhabdovirus, complete genome

NC_001989.1| Bovine respiratory syncytial virus, complete genome

NC_001921.1| Canine distemper virus, complete genome

NC_001906.2| Hendra virus, complete genome

NC_001803.1| Respiratory syncytial virus, complete genome

NC_001781.1| Human respiratory syncytial virus, complete genome

NC_001652.1| Infectious hematopoietic necrosis virus, complete genome

NC_002023.1| Influenza A virus (A/Puerto Rico/8/34(H1N1)) segment 1, complete sequence

NC_002022.1| Influenza A virus (A/Puerto Rico/8/34(H1N1)) segment 3, complete sequence

NC_002021.1| Influenza A virus (A/Puerto Rico/8/34(H1N1)) segment 2, complete sequence

NC_002019.1| Influenza A virus (A/Puerto Rico/8/34(H1N1)) segment 5, complete sequence

NC_002018.1| Influenza A virus (A/Puerto Rico/8/34(H1N1)) segment 6, complete sequence

NC_002017.1| Influenza A virus (A/Puerto Rico/8/34(H1N1)) segment 4, complete sequence

NC_006307.1| Influenza C virus segment 1, partial sequence
