# Supplementary material for: Patterns of Evolution and Host Gene Mimicry in Influenza and Other RNA Viruses
Source: PLoS Pathog. 2008 Jun 6;4(6):e1000079. doi: 10.1371/journal.ppat.1000079 (PMC2390760; doi:10.1371/journal.ppat.1000079)
Supplement: Text S3 — List of Human RNA viral genomes. (0.03 MB DOC) [file ppat.1000079.s003.doc]

**Supplementary Information 3: List of Human RNA viral genomes**

Human RNA+ viruses:

NC_001943.1| Human astrovirus, complete genome

NC_002640.1| Dengue virus 4, complete genome

NC_001477.1| Dengue virus 1, complete genome

NC_001475.2| Dengue virus 3, complete genome

NC_001474.2| Dengue virus 2, complete genome

NC_002058.3| Poliovirus, complete genome

NC_009825.1| Hepatitis C virus genotype 4, genome

NC_009824.1| Hepatitis C virus genotype 3, genome

NC_001959.2| Norwalk virus, complete genome

NC_001430.1| Human enterovirus D, complete genome

NC_009996.1| Human rhinovirus C, complete genome

NC_009827.1| Hepatitis C virus genotype 6, complete genome

NC_009826.1| Hepatitis C virus genotype 5, genome

NC_009823.1| Hepatitis C virus genotype 2, complete genome

NC_004102.1| Hepatitis C virus, complete genome

NC_009887.1| Human enterovirus 100, complete genome

NC_001472.1| Human enterovirus B, complete genome

NC_001489.1| Hepatitis A virus, complete genome

NC_001617.1| Human rhinovirus 89, complete genome

NC_001490.1| Human rhinovirus B, complete genome

NC_005831.2| Human coronavirus NL63, complete genome

NC_001897.1| Human parechovirus, genome

NC_001428.1| Human enterovirus C, complete genome

NC_001545.1| Rubella virus, complete genome

NC_001612.1| Human enterovirus A, complete genome

NC_006577.2| Human coronavirus HKU1, complete genome

NC_005147.1| Human coronavirus OC43, complete genome

Human RNA- virus:

NC_004291.1| Lymphocytic choriomeningitis virus segment L, complete sequence

NC_002020.1| Influenza A virus (A/Puerto Rico/8/34(H1N1)) segment 8, complete sequence

NC_002016.1| Influenza A virus (A/Puerto Rico/8/34(H1N1)) segment 7, complete sequence

NC_001498.1| Measles virus, complete genome

NC_004148.2| Human metapneumovirus, complete genome

NC_006312.1| Influenza C virus (C/Ann Arbor/1/50) segment 6, complete sequence

NC_006311.1| Influenza C virus (C/Ann Arbor/1/50) segment 5, complete sequence

NC_006310.1| Influenza C virus (C/Ann Arbor/1/50) segment 4, complete sequence

NC_006309.1| Influenza C virus (C/Ann Arbor/1/50) segment 3, complete sequence

NC_006308.1| Influenza C virus (C/Ann Arbor/1/50) segment 2, complete sequence

NC_002200.1| Mumps virus, complete genome

NC_007382.1| Influenza A virus (A/Korea/426/68(H2N2)) segment 6, complete sequence

NC_007381.1| Influenza A virus (A/Korea/426/68(H2N2)) segment 5, complete sequence

NC_007380.1| Influenza A virus (A/Korea/426/68(H2N2)) segment 8, complete sequence

NC_007378.1| Influenza A virus (A/Korea/426/68(H2N2)) segment 1, complete sequence

NC_007377.1| Influenza A virus (A/Korea/426/68(H2N2)) segment 7, complete sequence

NC_007376.1| Influenza A virus (A/Korea/426/68(H2N2)) segment 3, complete sequence

NC_007375.1| Influenza A virus (A/Korea/426/68(H2N2)) segment 2, complete sequence

NC_007374.1| Influenza A virus (A/Korea/426/68(H2N2)) segment 4, complete sequence

NC_007373.1| Influenza A virus (A/New York/392/2004(H3N2)) segment 1, complete sequence

NC_007372.1| Influenza A virus (A/New York/392/2004(H3N2)) segment 2, complete sequence

NC_007371.1| Influenza A virus (A/New York/392/2004(H3N2)) segment 3, complete sequence

NC_007370.1| Influenza A virus (A/New York/392/2004(H3N2)) segment 8, complete sequence

NC_007369.1| Influenza A virus (A/New York/392/2004(H3N2)) segment 5, complete sequence

NC_007368.1| Influenza A virus (A/New York/392/2004(H3N2)) segment 6, complete sequence

NC_007367.1| Influenza A virus (A/New York/392/2004(H3N2)) segment 7, complete sequence

NC_007366.1| Influenza A virus (A/New York/392/2004(H3N2)) segment 4, complete sequence

NC_003461.1| Human parainfluenza virus 1 strain Washington/1964, complete genome

NC_003443.1| Human parainfluenza virus 2, complete genome

NC_002211.1| Influenza B virus RNA 8, complete sequence

NC_002210.1| Influenza B virus RNA 7, complete sequence

NC_002209.1| Influenza B virus RNA 6, complete sequence

NC_002208.1| Influenza B virus RNA 5, complete sequence

NC_002207.1| Influenza B virus RNA 4, complete sequence

NC_002206.1| Influenza B virus RNA-3, complete sequence

NC_002205.1| Influenza B virus RNA-2, complete sequence

NC_002204.1| Influenza B virus RNA 1, complete sequence

NC_001781.1| Human respiratory syncytial virus, complete genome

NC_002023.1| Influenza A virus (A/Puerto Rico/8/34(H1N1)) segment 1, complete sequence

NC_002022.1| Influenza A virus (A/Puerto Rico/8/34(H1N1)) segment 3, complete sequence

NC_002021.1| Influenza A virus (A/Puerto Rico/8/34(H1N1)) segment 2, complete sequence

NC_002019.1| Influenza A virus (A/Puerto Rico/8/34(H1N1)) segment 5, complete sequence

NC_002018.1| Influenza A virus (A/Puerto Rico/8/34(H1N1)) segment 6, complete sequence

NC_002017.1| Influenza A virus (A/Puerto Rico/8/34(H1N1)) segment 4, complete sequence

NC_006307.1| Influenza C virus segment 1, partial sequence
